# The Impact of Bias Mitigation on Fairness and Accuracy in Automated Skin Lesion Classification

**DOI:** 10.1007/s41666-026-00252-w

**Published:** 2026-09-26

**Authors:** Matheus B. Rocha, Renato A. Krohling

**Affiliations:** 1 Labcin - Nature-inspired Computing Lab, Federal University of Espirito Santo, Vitoria, 29075-910 Brazil; 2 Graduate Program in Computer Science, Federal University of Espirito Santo, Vitoria, 29075-910 Brazil

**Keywords:** Debiasing, Fairness, Skin lesion, Clinical metadata, Multimodal fusion

## Abstract

To the best of our knowledge, existing benchmarks for bias mitigation in skin cancer classification focus exclusively on image-based, leaving a gap in the tabular-multimodal setting, where image-derived probabilities are combined with structured clinical data. To address this gap, this paper proposes an experimental framework to benchmark and integrate multiple bias mitigation algorithms into automated skin cancer classification pipelines based on a Mixed Fusion architecture. Using a multimodal fusion architecture originally developed for interpretability, we extend the framework to assess diagnostic bias across image data and metadata. Experiments were conducted on three datasets: PAD-UFES-20, HIBA, and MIDAS, incorporating sensitive demographic attributes to enable a fairness evaluation. Bias mitigation techniques were systematically applied at the pre-processing, in-processing, and post-processing stages, as well as in combination, using Multi-layer Perceptron (MLP), K-Nearest Neighbors (KNN), and Decision Tree (DT) classifiers. Performance and fairness were jointly analyzed using multiple metrics. According to the A-TOPSIS ranking, the MLP achieved the best results on DB-PAD-UFES-20, with a Balanced Accuracy (BACC) of 0.8313 and Statistical Parity (SP) of 0.0050 for gender and 0.0580 for Fitzpatrick skin type. On DB-HIBA, KNN ranked first, reaching a BACC of 0.7922 and SP of 0.0047 for gender. For DB-MIDAS, MLP again performed best, achieving a BACC of 0.6911 and SP of 0.0096 for gender. Post-processing methods consistently demonstrated effectiveness across datasets. By providing a methodological foundation with baseline results, this work paves the way for future research to develop more reliable clinical diagnosis that incorporate other bias mitigation techniques.

## Introduction

According to the World Health Organization (WHO) [1], cancer is projected to become the leading cause of mortality in the 21st century, with skin cancer being one of the types with the highest global incidence, which reinforces the need for effective methods for its early detection. In Brazil, according to the National Cancer Institute (INCA), skin cancer accounts for approximately *30%* of all malignant tumors registered in the country. For the period from 2023 to 2025, the occurrence of around 700 thousand new cancer cases is estimated, with non-melanoma skin cancers being the most frequent, representing *29.9%* of diagnoses in men and *32.7%* in women [2].

In recent decades, computer-aided diagnosis (CAD) systems have been developed with the aim of optimizing, standardizing, and accelerating the analysis of medical images, particularly to support cancer detection. These systems may contribute to the earlier identification of suspicious alterations and, consequently, to the more timely initiation of treatment. In this context, applications have been carried out across different imaging modalities, including magnetic resonance imaging (MRI), microscopic images for leukocyte detection, and images acquired through mobile devices, such as in the case of skin cancer [3–5].

These advances have been driven by improvements in feature extraction methods and by the integration of information from different modalities, such as clinical and demographic data, whose inclusion has, on average, improved performance [6, 7]. Recent discussions in the literature reinforce this trend and highlight the potential of multimodal fusion to enhance the accuracy and robustness of models [8].

Although promising, many of these models are considered black boxes, which hinders interpretability and the understanding of the factors influencing their predictions. Their high predictive power is accompanied by a lack of transparency that represents a risk in the context of medical diagnosis, where trust, accountability, and the ability to understand errors play a fundamental role [9, 10]. This trade-off between interpretability and predictive performance has been widely discussed in the literature, and defining an appropriate balance between these dimensions remains an open problem [11, 12]. In this direction, Ferreira and Couto [13] propose an explainable machine learning framework for the classification of breast lesions based on cytomorphometric data, whose compatibility with telemedicine and digital cytology workflows supports its integration into clinical practice and facilitates the transition from reactive to proactive oncologic care.

In parallel with the issue of interpretability, the strategy adopted for multimodal data integration also directly influences both model performance and their explainability. In the literature, this integration is predominantly performed at the intermediate stage of the classification process, an approach known as middle fusion [6, 7, 14]. Although this strategy enables the combination of more abstract representations from different modalities, it makes it more difficult to identify the features that effectively contributed to the diagnosis produced by the model. In contrast, alternative strategies such as early fusion and late fusion have also been employed, each with specific properties and methodological implications [15, 16]. In this context, with the aim of reconciling predictive performance and interpretability, Lima et al. [17] proposed the Mixed Fusion architecture, inspired by the clinical reasoning adopted by healthcare professionals and designed to enhance interpretability in deep multimodal fusion models.

Mixed Fusion also stands out for enabling interpretability and fairness analyses in multimodal scenarios, overcoming the limitation of models that provide only probability estimates as the final output. By combining tabular and image data, the architecture not only improves diagnostic performance but also establishes a more suitable framework for investigating critical open issues in the field, such as explainability, bias, and differential behavior across patient subgroups.

In light of these contributions, there is a growing need to understand how biases may manifest in model outputs and how mitigation techniques can operate in such scenarios. This motivation is reinforced by the significant increase in studies dedicated to investigating bias in artificial intelligence models, as well as to the development of strategies for its mitigation [18–20]. In this context, Raposo [21] discusses algorithmic identification failures in the field of facial recognition from different perspectives, identifying as central causes the probabilistic nature of the technology, its black-box character, and the presence of algorithmic bias, while also highlighting the possible legal consequences of such failures across different scenarios.

A variety of architectures and techniques have been proposed to address different fairness and robustness requirements. In this context, studies focusing on fairness in medical imaging have gained prominence in the literature due to the ethical and operational challenges inherent to the clinical domain [22].

Among these works, there is a growing focus on mitigating biases associated with skin tone in models applied to the classification of skin lesions. Aayushman et al. [23] investigated textual embeddings to align clinical information with image patches and analyzed model behavior across different skin tones. Park et al. [24] proposed a contrastive learning–based approach that integrates images and textual clinical data, aiming to reduce disparities arising from variations in skin tone.

Corbin and Marques [25] investigated joint regularization techniques and the mixing of synthetic images to mitigate bias across groups defined by skin tone, evaluating both performance and fairness with the support of Gradient-weighted Class Activation Mapping (Grad-CAM). Paxton et al. [26] developed an algorithm aimed at reducing skin tone–related bias in skin lesion classification by decreasing the influence of model channels associated with skin tone and focusing on regions relevant to the lesion.

Dang et al. [27] conducted a systematic comparison of post-processing debiasing methods, highlighting the advantages and limitations of each technique and analyzing the trade-offs between group fairness and predictive performance, as well as among different notions of fairness. Feldman and Peake [28] presented a mitigation pipeline that integrates pre-processing, in-processing, and post-processing methods, leveraging the strengths of each approach. However, the experiments conducted by the authors were restricted to a single dataset and did not involve applications in the medical domain.

Zong et al. [29] introduced a benchmark called MEDFAIR, in which they investigated different in-processing bias mitigation techniques across several skin cancer datasets, evaluating fairness from multiple perspectives and presenting recommendations for different medical application scenarios while considering distinct ethical principles. Their results indicated that state-of-the-art bias mitigation algorithms did not produce substantial fairness improvements when compared to the empirical risk minimization (ERM) strategy. More recently, Bayasi et al. [30] proposed the Debiasify model, which leverages a new knowledge distillation loss function throughout the network to induce the learning of representations that are more robust to bias, with the potential to simultaneously improve the performance of the most disadvantaged group and the overall accuracy across different datasets.

In addition to approaches specifically focused on image-based skin cancer classification, recent studies have also investigated bias mitigation strategies in other medical data contexts. In this regard, Goswami et al. [31] proposed FairMed-XGB, a multi-metric framework optimized through Bayesian methods, aimed at detecting and mitigating predictive bias related to the sensitive variable gender, while simultaneously preserving model performance and transparency. The approach incorporates a fairness-oriented loss function that combines the Statistical Parity Difference (SPD), Theil Index (TI), and Wasserstein Distance (WD) metrics, jointly optimized through Bayesian search within an XGBoost classifier.

In this study, we propose a fairness investigation pipeline that integrates multiple bias mitigation stages in automated skin cancer classification models. The use of Mixed Fusion enables the application of bias mitigation techniques at different stages of the model, allowing a comprehensive analysis of which strategies are more effective in mitigating inequalities in medical data related to skin cancer using tabular data and sensitive attributes. The debiasing approaches were evaluated regarding their impact on predictions and fairness metrics, being applied in combination with three external classifiers: a Multi-layer Perceptron (MLP), K-Nearest Neighbors (KNN), and a Decision Tree (DT). The results were analyzed using both performance and fairness metrics and subsequently integrated through the A-TOPSIS algorithm [32] to obtain a global ranking of the approaches.

Although previous studies have investigated bias mitigation strategies in image-based skin cancer classification, as far we know, no systematic examination has been conducted to assess how the three stages of bias mitigation, pre-processing, in-processing, and post-processing, individually and in combination, affect models applied to medical data, particularly in the context of tabular data derived from multimodal architectures. The Mixed Fusion architecture enables this analysis, as it allows the application of all three categories of mitigation techniques throughout the modeling pipeline, combining probabilities extracted from images with structured clinical data from patients.

In summary, the main contributions of this article are:

- The processing and creation of tabular data subsets derived from the PAD-UFES-20, HIBA, and MIDAS datasets for skin cancer diagnosis, resulting in the new DB-PAD-UFES-20, DB-HIBA, and DB-MIDAS datasets. These tabular datasets combine image-based probability outputs with clinical data and are prepared with a focus on bias mitigation, providing a foundation for fairness experiments in medical classification systems;
- A systematic investigation of representative bias mitigation techniques, with rigorous experiments involving three main approaches: pre-processing, in-processing, and post-processing, as well as their combinations. These approaches were applied to tabular data in the context of skin lesion diagnosis, offering a detailed analysis of the effectiveness of the selected techniques and their interactions, without claiming to exhaust the full range of methods within each category.
- A detailed comparative analysis of three classifiers, including two interpretable models, the Decision Tree and K-Nearest Neighbors, as well as a Neural classifier, with results demonstrating the superiority of the neural model in terms of performance.

The structure of the article is presented as in the following: in Section 2, the proposed pipeline, along with the methods and algorithms employed, are introduced; Section 3 presents the databases used in the experiments; in Section 4, the experimental configuration of the model, the results obtained, and the corresponding discussions are presented; finally, Section 5, concludes the article and outlines possible directions for future work.

## Methodology

### Fairness and Bias in Medical Data

In the medical context, diagnosis must not only be accurate and correct but also fair across different population groups. However, machine learning models are often trained on datasets that incorporate different forms of bias, originating, for example, from non-representative data collection processes, underrepresentation of minority groups, and incomplete records of clinical and socioeconomic information, among other factors [33, 34]. As a consequence, these systems may reproduce or amplify preexisting inequalities, compromising both the reliability of predictions and the fairness of their clinical application, even in scenarios where overall accuracy appears to be high.

The unequal representation of demographic subgroups in datasets is a recurrent source of bias in image-based diagnostic models. In the case of skin cancer, many of these datasets are predominantly composed of images from individuals with lighter skin tones, corresponding to Fitzpatrick skin types I to III, which may compromise model performance when applied to individuals with darker skin, classified as skin types IV to VI, increasing the risk of incorrect or delayed diagnoses [33]. In addition to skin tone, variations in the incidence of certain cancer types between men and women indicate that sex also constitutes a relevant sensitive attribute in this context [35].

In this context, the analysis of fairness and bias in medical data becomes essential for the development of safer, more transparent, and more appropriate models for sensitive decision-making scenarios. The main strategies adopted for this purpose can be grouped into three categories: pre-processing, in-processing, and post-processing. These approaches aim to reduce the influence of biased information and, consequently, mitigate the bias embedded in the models, promoting greater fairness in predictions. However, the application of these techniques may introduce a trade-off between fairness and performance, since bias reduction may, in certain scenarios, be accompanied by a decrease in model performance [36, 37].

### Bias Mitigation Strategies

#### Pre-Processing

Pre-processing bias mitigation methods are characterized by being model- and data-agnostic, with the objective of performing modifications on the dataset to remove undesired biases. The idea is to obtain a modified dataset that improves fairness or to apply techniques and rules that remove or compensate for underrepresented groups in the data. A model trained on such data will therefore be exposed to a more representative data distribution, resulting in a fairer model [19].

There are several categories of methods applicable to tabular data for bias mitigation in the pre-processing stage. Among them, Massaging [38] stands out, which involves altering instance labels in the dataset to remove disparities and, consequently, discrimination. The Reweighing [39], in which weights are assigned to the dataset to control the influence of biases, and Sampling [40], which involves under-sampling and over-sampling groups of a protected variable with respect to the target variable. Collectively, these methods aim to modify the data distribution in order to improve the representation of minority groups and adjust the individual contribution of samples during model training.

Among the existing methods, the Debiaser for Multiple Variables (DEMV) [41] was employed. DEMV is a sampling-based method designed to increase fairness in datasets with binary or categorical data containing one or more sensitive variables. It considers all possible combinations of sensitive variable values and class labels, thereby forming sensitive groups whose objective is to be balanced. For each group, the expected size ($W_{exp}$) and the observed size ($W_{obs}$) are computed as follows:

1 $$\begin{aligned} W_{exp}&= \frac{\big |\{X \in D| S = s\}\big |}{\big |D\big |} * \frac{\big |\{X \in D| L = l\}\big |}{\big |D\big |} \end{aligned}$$

2 $$\begin{aligned} W_{obs}&= \frac{\big |\{X \in D| S = s \wedge L = l \}\big |}{\big |D\big |} \end{aligned}$$

where *S = s* represents a condition on the value of the sensitive variables and *L = l* represents a condition on the label value, and *D* denotes the dataset. After this calculation, a ratio between these two values ($W_{exp}/W_{obs}$) is computed; if it is less than 1, the element is removed, and if it is greater than 1, an element is added until this ratio becomes $W_{exp}/W_{obs} = 1$.

#### In-Processing

In-processing methods aim to mitigate bias during model training. In contrast to pre-processing approaches, these techniques depend directly on the architecture employed and on the behavior of the data during optimization, making use of new or adapted algorithms, or alternatively, the addition of a regularization term to the loss function.

Several strategies can be employed in this context. One category involves the introduction of fairness-related constraints or penalties during training, modifying the objective function to balance predictive performance and bias reduction [42]. Another line of methods relies on learning algorithms designed to seek explicit trade-offs between accuracy and fairness metrics. There are also adversarial approaches applied to the fairness problem [43], in which two models are trained simultaneously: the first is trained to predict the true label, while the second attempts to infer the sensitive variable from the predictions of the first, thereby encouraging the removal of information correlated with the protected attribute. Another approach consists of methods based on adapted loss functions [31, 44], which introduce modifications to the optimization function, either by replacing it with an alternative or by adding regularization terms, in order to explicitly incorporate fairness criteria into the training process.

Among the available methods, the adversarial approach based on an Adversarial Variational Autoencoder (VAE-Adv) was used. This choice was motivated by recent literature that employs similar techniques in the context of medical data [45, 46]. The operation of the method can be described as follows: the input vector *X* is processed by the encoder, which projects it into a latent representation space parameterized by a mean and a standard deviation. A sample from this latent space is obtained through a sampling function and is then passed to the decoder, which is responsible for reconstructing the original data $\hat{X}$. In parallel, an adversarial network receives the latent representation as input and is trained to predict the sensitive variable, thereby encouraging the model to remove information correlated with the protected attribute at this stage.

The loss function of the VAE-Adv is composed of three terms. The first corresponds to the reconstruction error, given by:

3 $$\begin{aligned} \mathcal {L}_{\text {recon}} = \frac{1}{K}\sum _{i=1}^{K} ||X - \hat{X}||_{2}^{2} \end{aligned}$$

where *X* represents the input data, $\hat{X}$ the reconstruction generated by the decoder, and *K* the batch size. The second term is the Kullback–Leibler divergence (KLD), described by:

4 $$\begin{aligned} \mathcal {L}_{\text {KLD}} = D_{KL}\big (q_{\theta }(Z\mid X) \parallel p(Z)\big ) = - \frac{1}{2}\sum _{j=1}^{J}\Bigg [ 1 + \log (\sigma _{j}^{2}) - \mu _{j}^{2} - \sigma _{j}^{2} \Bigg ] \end{aligned}$$

in which *Z* is the latent representation, *θ* denotes the encoder parameters, and *μ* and *σ* are, respectively, the mean and standard deviation estimated by the encoder for each dimension *j* of the latent space. The third term is the adversarial loss, described by:

5 $$\begin{aligned} \mathcal {L}_{\text {Adv}} = -\sum _{i=1}^{n_{attr}} \bigg [s_i \log (\hat{s}_i) + (1-s_i) \log (1-\hat{s}_i)\bigg ] \end{aligned}$$

where $s_i$ is the true label of the sensitive variable, $\hat{s}i$ is the probability estimated by the discriminator, and *n* *attr* corresponds to the number of sensitive attributes. The overall loss function is given by:

6 $$\begin{aligned} \mathcal {L}_{\text {VAE}_{\text {Adv}}} = \mathcal {L}_{\text {recon}} + \beta \cdot \mathcal {L}_{\text {KLD}} - \lambda \cdot \mathcal {L}_{\text {Adv}} \end{aligned}$$

where $\mathcal {L}{\text {recon}}$ corresponds to the loss term associated with input reconstruction, $\mathcal {L}{\text {KLD}}$ is the Kullback–Leibler divergence between the posterior latent distribution and the standard normal distribution, and $\mathcal {L}_{\text {Adv}}$ is the adversarial term, with binary cross-entropy being employed. The hyperparameters *β* and *λ* are scalar values used to balance the contribution of the KLD and adversarial terms, ensuring that they operate at the same magnitude as the reconstruction error during model optimization.

#### Post-Processing

Post-processing methods are applied directly to the predictions of already trained models, without the need to retrain the classifier, and are recommended in scenarios in which access to the complete model is not available or when its modification is not feasible. An important characteristic of these techniques is that they are agnostic to the previously trained model [27].

Among existing method categories, Classifier Correction [47] stands out, adjusting the probabilities associated with the positive class to reduce disparities between groups. Another method is Output Correction [48], in which the predicted labels are modified based on predefined rules to correct potential biases by adjusting the confidence levels associated with the decisions.

The post-processing methods considered in this study is the MLDebiaser [49], which mitigates bias in predictions with respect to the sensitive variable for each demographic group. The method requires the target label vector *y* as input and calibrates the outputs of a previously trained classifier by solving a convex optimization problem subject to a statistical parity constraint. This technique is applicable to both binary and multi-class classification tasks.

The procedure operates as follows: given the target label vector *y* and the predictions $f(x_i)$ of the classifier, the method seeks to adjust the new outputs $h(x_i)$ in such a way that they remain as close as possible to $f(x_i)$, while imposing statistical parity constraints. To achieve this, it solves the following optimization problem:

7 $$\begin{aligned} \min _{0 \le h(x_i) \le 1} \quad \sum _{x_i \in S} (\gamma /2)h(x_i)^2 - f(x_i)h(x_i)\end{aligned}$$

8 $$\begin{aligned} \text {s.t. } \quad \forall k \in [K] : \left| \sum _{x_i \in S_k} (h(x_i) - \rho ) \right| \le \epsilon _k \end{aligned}$$

where $S_k$ denotes the subset of samples belonging to group *k* defined by the sensitive attribute, *K* is the total number of groups, $\epsilon _k = |S_k|,\epsilon /2$ with $\epsilon \ge 0$ representing the desired tolerance for violations of statistical parity, *γ> 0* is a regularization hyperparameter that controls the degree of decision randomization, and $\rho \in [0,1]$ is a hyperparameter tuned during validation.

### Experimental Framework

**Fig. 1:**
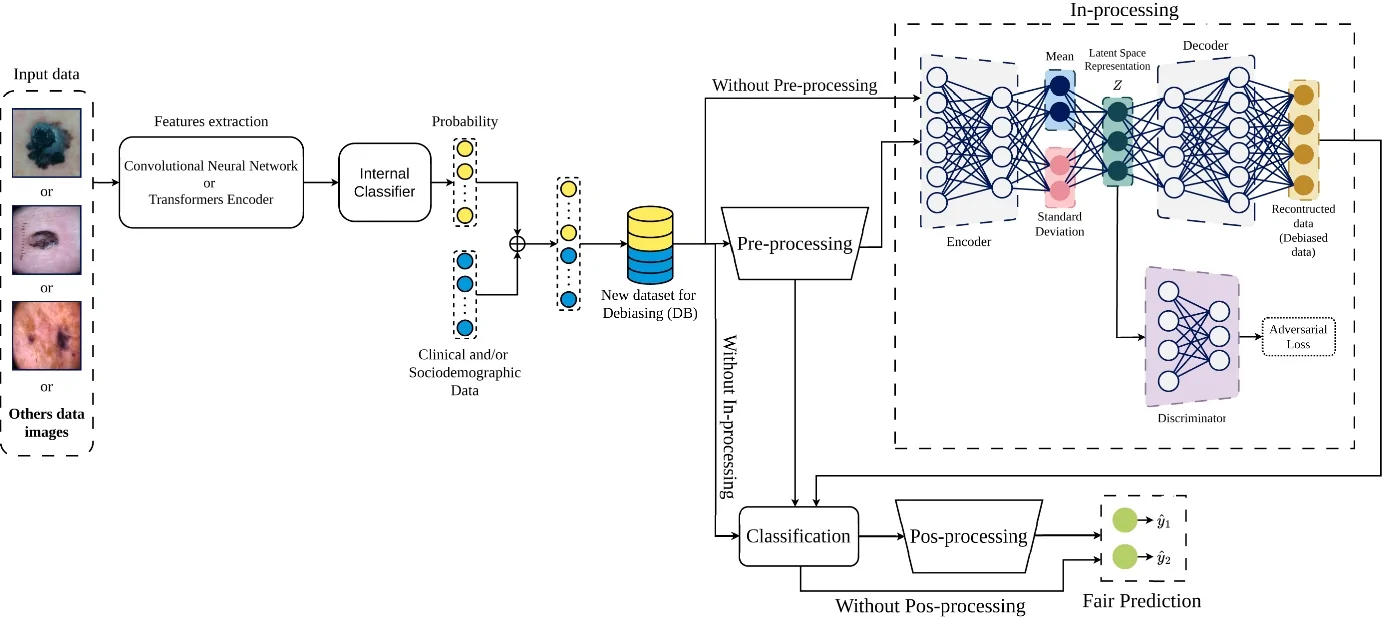
The complete architecture of the pipeline, divided into two phases. In Phase 1, a Mixed Fusion [17] architecture is employed to obtain probability estimates, which are then concatenated with the clinical data. Phase 2 varies according to the bias mitigation technique adopted. In the pre-processing method (Pre), the data are transformed through the mitigation step and subsequently fed into the classification network. In the in-processing method (In), the data are passed through an Adversarial Variational Autoencoder, and the reconstructed representations are used as input to the classification network. In the post-processing method (Post), the prediction generated by the classification network is adjusted using a mitigation technique applied after inference

The proposed pipeline, illustrated in Fig. 1, aims to evaluate how different bias mitigation approaches perform on medical data, and its construction is defined by two distinct phases. In Phase 1, the Mixed Fusion architecture is employed, in which images are processed by the network to obtain class-associated probability estimates. Each resulting probability is then concatenated with the corresponding clinical data, giving rise to a new dataset oriented toward bias mitigation, which is subsequently used in Phase 2. Phase 2 corresponds to the stage in which bias mitigation techniques are applied, resulting in eight distinct approaches. The first, None, consists of the absence of any mitigation technique. In the Pre-processing approach, the DEMV pre-processing technique is applied prior to the classification process. In the In-processing approach, the in-processing method based on the VAE-Adv is employed, and its reconstructions are used as input to the classifier. In the Post-processing approach, the classifier predictions are adjusted using the MLDebiaser post-processing method.

The remaining approaches consist of combinations of these techniques. In the PI (Pre-processing + In-processing) combination, the data processed by DEMV are used as input to the VAE-Adv, and its reconstructions are passed to the classifier. In the PP (Pre-processing + Post-processing) combination, the data transformed by DEMV are classified, and the predictions are subsequently adjusted by MLDebiaser. In the IP (In-processing + Post-processing) combination, the data first pass through the VAE-Adv, are classified, and have their predictions adjusted by MLDebiaser. Finally, the PIP (Pre-processing + In-processing + Post-processing) approach integrates all techniques, applying DEMV prior to the VAE-Adv, whose reconstructions feed the classifier, and the predictions are then adjusted by MLDebiaser.

## Data and Preprocessing

### Datasets

#### DB-PAD-UFES-20

The PAD-UFES-20 dataset [50] contains clinical images of skin lesions and clinical data from patients, collected between 2018 and 2019. The dataset comprises information from 1,373 patients associated with 1,641 unique lesions, resulting in a total of 2,298 clinical images captured using mobile devices.

 These images were processed by the Mixed Fusion model to generate the image probabilities. Subsequently, these probabilities were concatenated with the corresponding clinical data, forming a subset named Debiasing-PAD-UFES-20 (DB-PAD-UFES-20).

A limitation of the PAD-UFES-20 dataset concerns the pre-processing procedure, specifically the removal of irrelevant columns and missing data. Only lesions that underwent biopsy had complete clinical data. After this filtering step, 11 clinical variables were retained: age, sex, Fitzpatrick scale, lesion diameter (horizontal and vertical), itching, growth, pain, change, bleeding, and elevation. For the categorical variables, one-hot encoding was applied. As a result, the clinical data were transformed into 23 encoded features. Using only samples with complete clinical data, the number of lesions was reduced to 1.474, of which 385 are benign and 1,089 are malignant, representing *26.12%* and *73.88%* of the samples, respectively. The variables included in the DB-PAD-UFES-20 dataset are listed in Table 1.

**Table 1:** Characteristics of the DB-PAD-UFES-20 dataset, organized by data source and variable type (continuous, categorical, binary)

Modality	Feature	Type	Sensitive Attribute
Image Information	Prob. of being benign	Continuous	No
	Prob. of being malignant	Continuous	No
Clinical Information	Age	Continuous	No
	Gender	Categorical	Yes
	Fitzpatrick scale	Binary (I-III and IV-VI)	Yes
	Horizontal diameter	Continuous	No
	Vertical diameter	Continuous	No
	Itch	Binary	No
	Grew	Binary	No
	Hurt	Binary	No
	Changed	Binary	No
	Bleed	Binary	No
	Elevation	Binary	No

**Fig. 2:**
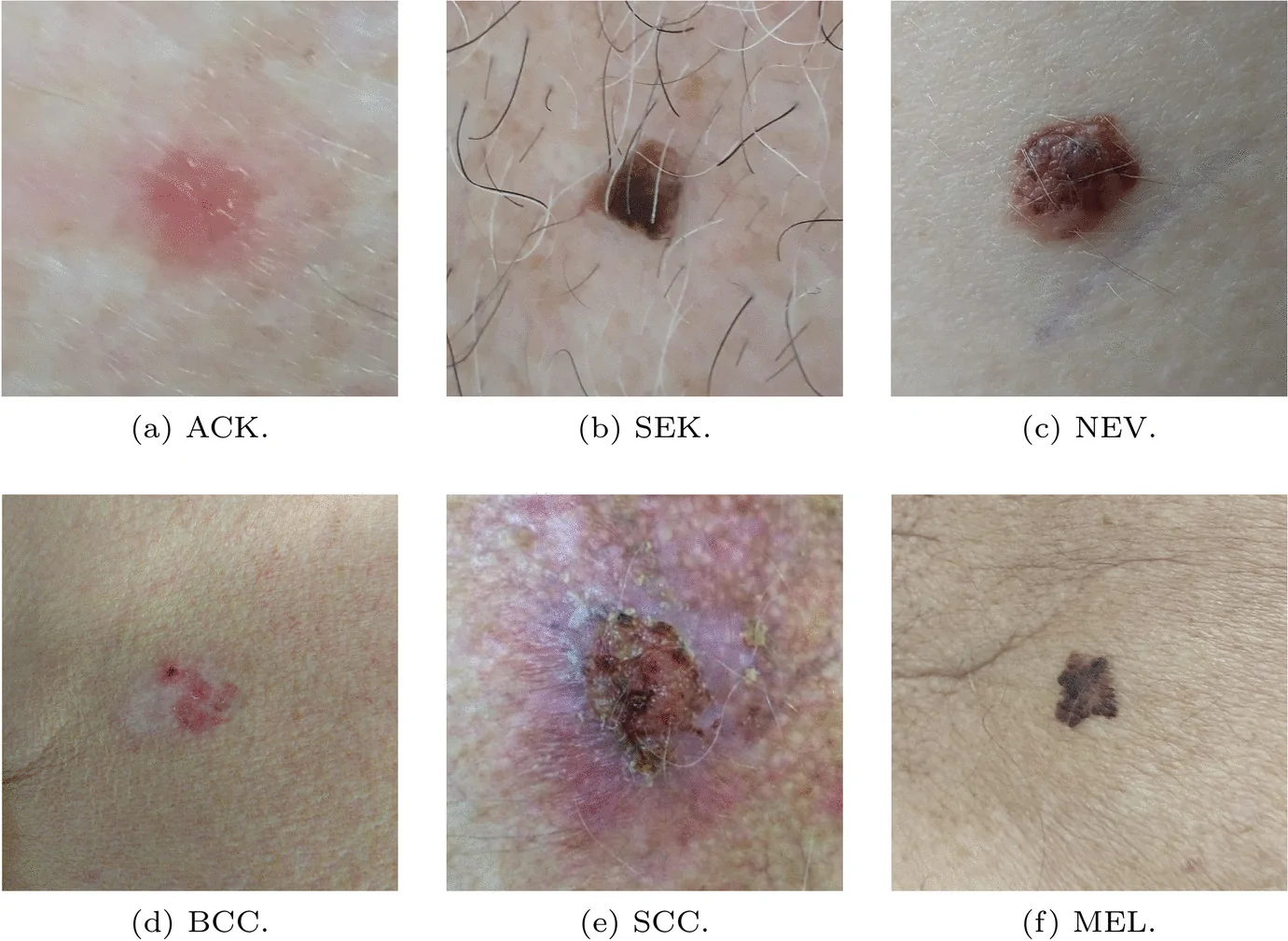
Examples of images present in the PAD-UFES-20 dataset

Considering the distribution by lesion type, the dataset includes three malignant classes: Basal Cell Carcinoma (BCC) with 845 samples, Squamous Cell Carcinoma (SCC) with 192 samples, and Melanoma (MEL) with 52 samples. The benign lesions are distributed across three classes: Actinic Keratosis (ACK) with 273 samples, Melanocytic Nevus (NEV) with 72 samples, and Seborrheic Keratosis (SEK) with 40 samples. Figure 2 shows an example of the six most frequent skin lesions in the PAD-UFES-20 dataset.

#### DB-HIBA

Introduced by Ricci Lara et al. [51], the HIBA dataset contains clinical images, dermoscopic images, and clinical data from patients at the Hospital Italiano de Buenos Aires, collected between 2019 and 2022. HIBA comprises information from 623 patients, totaling 1,616 images, of which 1,270 correspond to dermoscopic images acquired with polarized contact and 346 are clinical images. These images are associated with 1,246 lesions belonging to the most frequent diagnoses observed at the institution.

Due to the larger volume of available images, only the dermoscopic subset was used in the experiments. These images were processed by the Mixed Fusion model, from which classification probabilities were obtained. Subsequently, these probabilities were concatenated with the corresponding clinical data, resulting in a derived subset referred to as Debiasing-HIBA (DB-HIBA).

The DB-HIBA dataset underwent an additional pre-processing step, in which clinical attributes with a high number of missing values or considered not relevant for medical diagnosis were removed. After this filtering, six clinical variables were retained: approximate patient age, anatomical region of the lesion, family history of melanoma, Fitzpatrick skin tone scale, personal history of melanoma, and sex. For the categorical variables, one-hot encoding was used for their representation. In total, the complementary data were transformed into 15 encoded features. Samples presenting missing values in any sensitive variable were also excluded, resulting in the removal of 107 lesions. At the end of the process, the dataset used in this study comprised 1,163 dermoscopic lesions accompanied by their corresponding clinical data. Among these lesions, 664 are benign and 499 are malignant, corresponding to *57.1%* and *42.9%* of the samples, respectively. The variables included in DB-HIBA are listed in Table 2.

**Table 2:** Characteristics of the DB-HIBA dataset, organized by data source and variable type (continuous, categorical, binary)

Modality	Feature	Type	Sensitive Attribute
Image Information	Prob. of being benign	Continuous	No
	Prob. of being malignant	Continuous	No
Clinical Information	Age	Continuous	No
	Gender	Categorical	Yes
	Anatomic Location	Categorical	No
	Family history of MM	Binary	No
	Personal history of MM	Binary	No
	Fitzpatrick scale	Binary (I-III and IV)	No

**Fig. 3:**
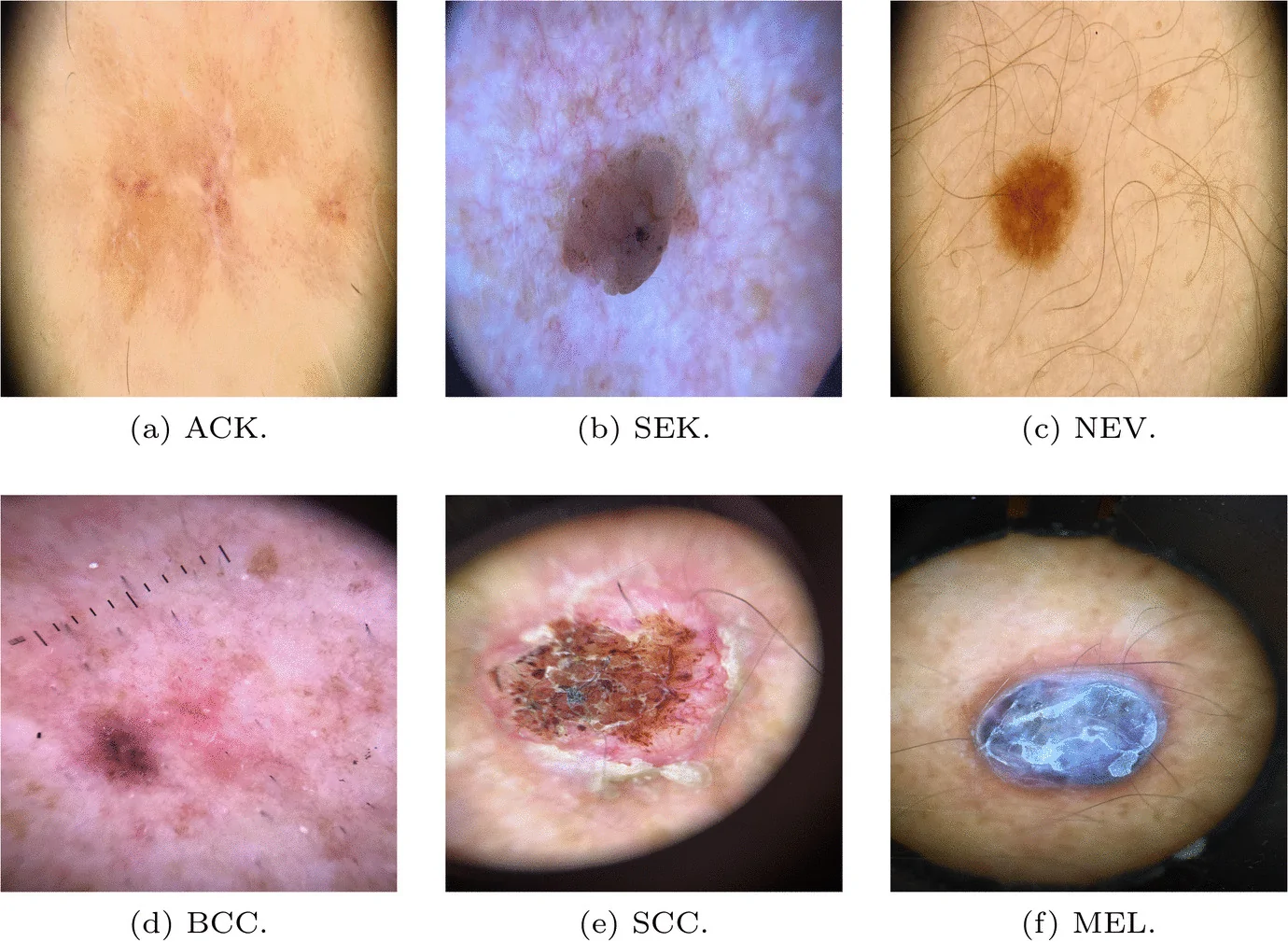
Examples of dermoscopic images of the six most frequent analyzed lesions in the HIBA dataset

Considering the distribution by lesion type, the dataset includes three malignant classes: Basal Cell Carcinoma (BCC) with 208 samples, Squamous Cell Carcinoma (SCC) with 107 samples, and Melanoma (MEL) with 184 samples. The benign lesions are distributed across seven classes: Melanocytic Nevus (NEV) with 496 samples, Seborrheic Keratosis (SEK) with 43 samples, Actinic Keratosis (ACK) with 42 samples, Dermatofibroma (DF) with 36 samples, Vascular Lesion (VASC) with 33 samples, Solar Lentigo (SL) with 13 samples, and Lichenoid Keratosis (LK) with 1 sample. Figure 3 shows an example of the six most frequent skin lesions in the HIBA dataset.

#### DB-MIDAS

The Melanoma Research Alliance Multimodal Image Dataset for AI-based Skin Cancer (MIDAS) [52] contains clinical images obtained at distances of 15 cm and 30 cm, dermoscopic images, and clinical data from patients at Stanford and the Cleveland Clinic. MIDAS comprises information from 796 patients, totaling 3,830 images associated with 1,290 unique lesions, of which 1,187 correspond to dermoscopic images, 1,198 to clinical images captured at 15 cm, 1,185 to clinical images captured at 30 cm, and 260 images submitted by patients.

Following the same procedure adopted for DB-HIBA, only the dermoscopy image subset was used in the experiments. These images were processed by the Mixed Fusion model, from which the classification probabilities were obtained. Subsequently, these probabilities were concatenated with the respective clinical data, resulting in a derived subset named Debiasing-MIDAS (DB-MIDAS).

In the original metadata, columns considered not relevant for medical diagnosis were removed, and the column midas_path was adopted as the target variable. After this filtering step, six clinical variables were retained: patient age and sex, lesion location, lesion height and width (in millimeters), and the Fitzpatrick skin tone scale. The categorical variable related to lesion location was encoded using one-hot encoding. In total, the complementary clinical data were transformed into 302 encoded features. The final dataset used in this study contains 913 dermoscopic lesions accompanied by their corresponding clinical data. Among these lesions, 441 are benign and 472 are malignant, corresponding to *48.3%* and *51.7%* of the samples, respectively. The variables included in DB-MIDAS are listed in Table 3.

**Table 3:** Characteristics of the DB-MIDAS dataset, organized by data source and variable type (continuous, categorical, binary)

Modality	Feature	Type	Sensitive Attribute
Image Information	Prob. of being benign	Continuous	No
	Prob. of being malignant	Continuous	No
Clinical Information	Age	Continuous	No
	Gender	Categorical	Yes
	Lesion location	Categorical	No
	Lesion length (mm)	Continuous	No
	Lesion width (mm)	Continuous	No
	Fitzpatrick scale	Binary (I-III and IV)	No

**Fig. 4:**
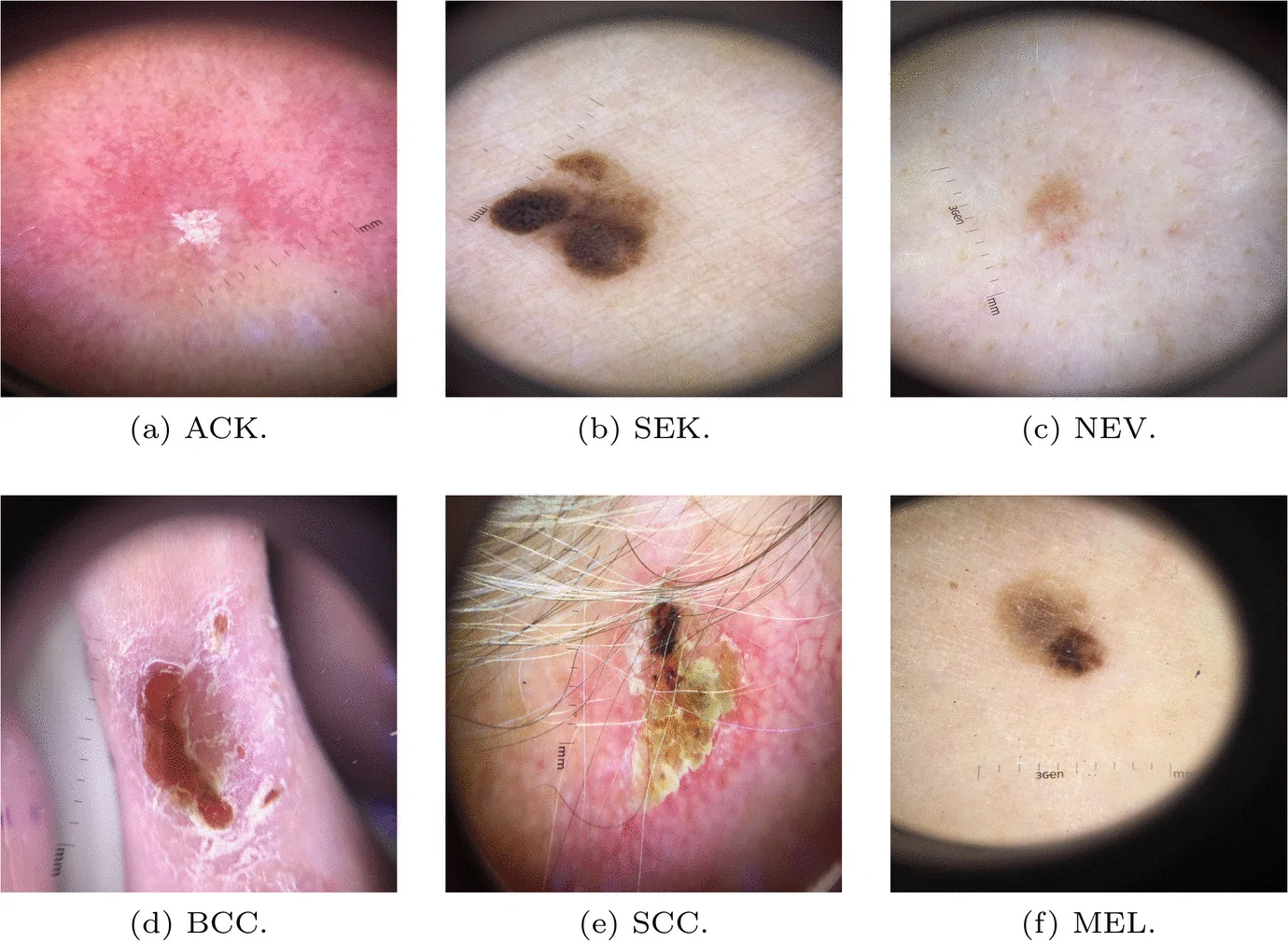
Examples of dermoscopic images of the six most frequent analyzed lesions in the MIDAS dataset

Considering the distribution by lesion type, the dataset includes three malignant classes: Basal Cell Carcinoma (BCC) with 202 samples, Squamous Cell Carcinoma (SCC) with 123 samples, and Melanoma (MEL) with 79 samples. The benign lesions are distributed across seven classes: Melanocytic Nevus (NEV) with 192 samples, Seborrheic Keratosis (SEK) with 79 samples, Actinic Keratosis (ACK) with 64 samples, Dermatofibroma (DF) with 16 samples, Hemangioma with 10 samples, Fibrous Papule (FP) with 6 samples, and Other with 142 samples. Figure 4 shows an example of the six most frequent skin lesions in the MIDAS dataset.

### Cross Validation

The data were partitioned using the holdout validation technique, in which 5/6 of the dataset were allocated for training and the remaining 1/6 reserved for testing. Additionally, the training data were further split using the cross-validation (CV) technique. This approach is employed to avoid methodological errors and consists of dividing the data into *k* equal parts, with (*k-1*) parts used for training and one part for validation. This process is repeated *k* times, changing the validation set at each iteration and ensuring that the entire dataset is covered. In the experiments, *k = 5* was used, a standard value available in the scikit-learn library [53]. For each training process, a new model is trained, preventing data contamination between the training and validation sets.

Cross-validation was employed in the initial stage of image data partitioning, which was used to obtain the predicted probabilities. In the subsequent stage, corresponding to the training of the external classifiers using the new dataset, the same partitions defined in the previous stage were reused. In this way, the fold designated for testing remained strictly held out, while the training set followed the previously established split, in order to preserve methodological consistency and ensure the integrity of the experimental evaluation. For all processes, the dataset splitting was carried out as follows: the data were first divided into training and test sets, with 80% allocated for training and 20% for testing. Subsequently, the subset designated for training was further partitioned into 80% for training and 20% for validation within each of the *k* folds.

Additionally, stratification was applied based on the lesion diagnosis and the corresponding sensitive variable in each dataset. This procedure ensures the preservation of the proportional distribution of classes and sensitive groups across all partitions, guaranteeing adequate sample availability for the evaluation of bias mitigation techniques and promoting consistency in the results obtained throughout the cross-validation iterations.

To ensure reproducibility and consistency across multiple experiments, a fixed random seed was used both for the split between the training and test sets and for the cross-validation (CV) divisions between the training and validation sets. This methodological approach aims to maintain uniformity in the selections made during each test iteration.

### Metrics

In medical contexts, the accuracy, consistency, and impartiality of a diagnosis are essential to ensure that treatment is administered appropriately and in a timely manner, especially in critical situations that require immediate intervention. This principle applies equally to the context of skin lesion classification, since certain lesions may exhibit malignant characteristics and therefore pose a significant risk to patient health. In this scenario, it is essential that the prediction process is not influenced by external variables irrelevant to the clinical outcome, in order to ensure the fairness and reliability of the system.

#### Performance

The performance results of the models were analyzed by considering the mean and standard deviation of the metrics for each combination. Five metrics widely used in the literature were employed: Accuracy (ACC), Balanced Accuracy (BACC), Precision, Recall, and F1-Score, which are defined as follows:

9 $$\begin{aligned} ACC = \frac{TP + TN}{TP + FP + TN + FN} \end{aligned}$$

10 $$\begin{aligned} BACC = \frac{\frac{TP}{TP + FN} + \frac{TN}{TN + TP}}{2} \end{aligned}$$

11 $$\begin{aligned} Recall = \frac{TP}{TP + FN} \end{aligned}$$

12 $$\begin{aligned} Precision = \frac{TN}{TN + FP} \end{aligned}$$

13 $$\begin{aligned} \textit{F1-score} = 2 \times \frac{Recall \times Precision}{Recall + Precision} \end{aligned}$$

where the variables *TP*, *TN*, *FP*, and *FN* represent, respectively, True Positive, True Negative, False Positive, and False Negative. These metrics are used to evaluate the effectiveness of the algorithm employed in the classification process, providing a detailed view of the model’s performance in terms of its ability to correctly identify malignant and benign lesions. Among the metrics considered, Balanced Accuracy (BACC) was regarded as the primary performance metric in the analysis of the experimental results due to its greater reliability in scenarios with imbalanced data.

#### Fairness

The fairness results of the models were analyzed by considering the mean and standard deviation of the metrics for each combination of techniques and classifiers. Although there is no universally accepted definition of which fairness metric is most appropriate across all contexts, the selection of metrics adopted in this study was grounded in recent literature [18, 19]. Four metrics widely used for evaluating group fairness were employed: Statistical Parity (SP), Disparate Impact (DI), Equal Opportunity Difference (EOD), and Average Odds Difference (AOD).

14 $$\begin{aligned} SP = P(\hat{y} = 1 \mid S = s_1) - P(\hat{y} = 1 \mid S = s_2) \end{aligned}$$

15 $$\begin{aligned} DI = \frac{P(\hat{y} = 1 \mid S = s_1)}{P(\hat{y} = 1 \mid S = s_2)} \end{aligned}$$

16 $$\begin{aligned} EOD = TPR_{S=s_1} - TPR_{S=s_2} \end{aligned}$$

17 $$\begin{aligned} AOD = \frac{1}{2}\Big [(FPR_{S=s_1} - FPR_{S=s_2}) + (TPR_{S=s_1} - TPR_{S=s_2})\Big ] \end{aligned}$$

where *TPR* and *FPR* represent, respectively, the True Positive Rate and the False Positive Rate. Among the defined metrics, Disparate Impact (DI) has a reference value of 1.0, indicating the absence of disparity between groups. For the remaining metrics (SP, EOD, and AOD), the reference value is 0.0, such that values closer to zero indicate greater fairness according to the metric considered.

### Implementation Details

**Fig. 5:**
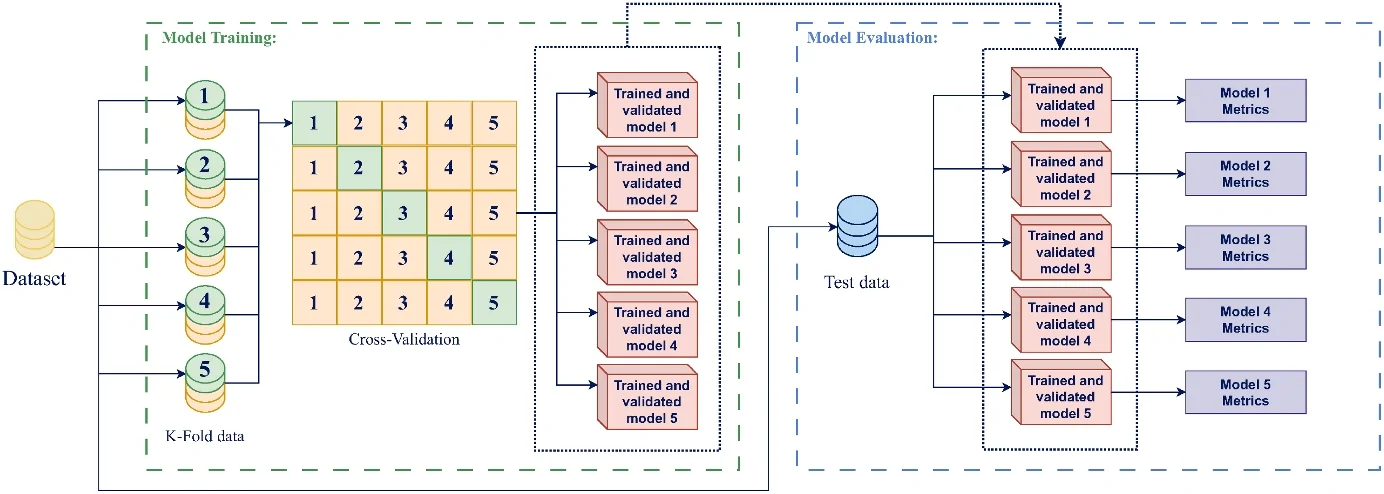
Diagram representing the experiment, displaying data split, model training, model evaluation, and results analysis steps that occurred for each model

The experiments were conducted as follows: initially, an internal classifier, based on Lima et al. [17], was trained to obtain image probability estimates, and the data were partitioned using using holdout validation and cross-validation (CV). The probabilities used in the subsequent stage were obtained from the validation subsets of each fold. At the end of this procedure, each sample in the development set had exactly one out-of-fold prediction, that is, a prediction generated by a model that had not been trained on that sample. These probabilities were then concatenated with the corresponding clinical data, resulting in a new dataset composed exclusively of tabular attributes. For the test set, the model corresponding to the fold with the best performance on the validation set, measured by balanced accuracy (BACC), was selected. This model was then used to extract the probabilities for the test set. Finally, these probabilities were concatenated with the clinical variables, forming the dataset used for training the downstream classifiers, while preserving the same previously defined partitions and keeping the test set strictly separated at all times. From this new dataset, the bias mitigation methods were applied. Figure 5 shows a diagram illustrating the data partitioning configuration used in the experiments and the steps performed to analyze the results for each type of model. In order to allow reproducibility of the results, the source code is available at: https://github.com/MatheusBecali/bias-fairness-skin-lesion.

Initially, hyperparameter tuning of the models was performed. Table 4 presents the search space used for this analysis in the PIP approach across all three datasets. These hyperparameters were adjusted with the aim of optimizing model performance. The search for the best combinations of hyperparameters was conducted using the training set. Subsequently, the best hyperparameter configuration found was used for training and evaluating the model. The hyperparameter tuning was performed through 2000 iterations using the Optuna framework [54] with the Tree-structured Parzen Estimator (TPE) sampler [55].

**Table 4:** Hyperparameter tuning for PIP

Hyperparameter	DB-PAD-UFES-20	DB-HIBA	DB-MIDAS
lr vae ($lr_{vae}$)	[0.00001 *∼* 0.01]	[0.00001 *∼* 0.005]	[0.00001 *∼* 0.005]
lr adv ($lr_{adv}$)	[0.000001 *∼* 0.001]	[0.00001 *∼* 0.02]	[0.00001 *∼* 0.02]
lambda adv ($\lambda _{adv}$)	[0.001 *∼* 1.0]	[0.01 *∼* 5.0]	[0.01 *∼* 5.0]
beta (*β*)	[0.001 *∼* 1.0]	[0.01 *∼* 10.0]	[0.01 *∼* 10.0]
batch size vae ($b_{size\_vae}$)	[8 *∼* 48]	[8 *∼* 48]	[8 *∼* 48]
optimizer vae ($opt_{vae}$)	[Adam, SGD]	[Adam, SGD]	[Adam, SGD]
batch size mlp ($b_{size\_mlp}$)	[16 *∼* 128]	[16 *∼* 128]	[16 *∼* 128]
optimizer mlp ($opt_{mlp}$)	[Adam, SGD]	[Adam, SGD]	[Adam, SGD]

Regarding the external classification network, three classifiers were evaluated: Multi-Layer Perceptron (MLP), K-Nearest Neighbors (KNN), and Decision Tree (DT). In the adopted configuration, the MLP comprises a hidden layer whose dimension corresponds to half the input size. To mitigate the risk of overfitting, batch normalization and a dropout rate of 0.1 were employed. The activation function utilized is the hyperbolic tangent (Tanh).

For the MLP external classifier, a fixed learning rate (*lr*) of $10^{-3}$ was employed for the optimizer. Additionally, a learning rate decay strategy was implemented with a decay factor of 0.1 if the model did not improve for a consecutive period of 20 epochs. An early stopping strategy was also adopted for both the VAE training and the external classifiers, interrupting the training process if no improvements were observed for 20 consecutive epochs in the classifiers or for 50 epochs in the case of the VAE.

For the KNN external classifier, the number of neighbors was fixed at *k = 3*, while for the DT the maximum depth was set to 3. These hyperparameters were selected through an exhaustive search, considering values in the range from 2 to 10. The final choice was based on the configuration that achieved the highest balanced accuracy (BACC) on the validation set.

## Results and Discussion

### Experimental Results

In this work, we investigated the performance of different bias mitigation stages using three fairness-oriented modified versions of the datasets PAD-UFES-20 [50], HIBA [51], and MIDAS [52], resulting in the DB-PAD-UFES-20, DB-HIBA, and DB-MIDAS. The best overall results for each group of experiments are highlighted in **bold**.

To obtain image classification probabilities, the ResNet-50 model was employed across all datasets. The generated probabilities were subsequently concatenated with the corresponding clinical data. The resulting data were then subjected to bias mitigation techniques. To acquire the results, experiments were conducted using three external classifiers selected for comparative purposes: Multi-Layer Perceptron (MLP), K-Nearest Neighbors (KNN), and Decision Tree (DT).

In the experiments conducted, the fairness analysis was focused on the concept of group fairness, understood as the absence of systematic favoritism toward any subgroup in the decision-making process of machine learning models, as defined by Saxena et al. [56]. In this context, the fairness evaluation considered two distinct groups: group *a*, composed of the underprivileged population, and group *b*, composed of the privileged population. The definition of these groups was determined by the sensitive variable available in each dataset used in the study.

In the DB-MIDAS and DB-HIBA datasets, the sensitive variable used for fairness evaluation was gender. In the case of the DB-PAD-UFES-20 dataset, due to the presence of all six Fitzpatrick scales, which were grouped as 0.0 for scales I–III and 1.0 for scales IV–VI, the fairness analysis was conducted based on two sensitive variables: gender and Fitzpatrick skin tone. Although the DB-HIBA and DB-MIDAS datasets include the Fitzpatrick variable, they do not contain patients classified in scales V and VI; therefore, the analysis was restricted to the gender variable.

For each dataset, the experiments followed the same methodology: initially, classification was performed without the application of bias mitigation techniques, thereby establishing a baseline for comparison. Subsequently, three categories of mitigation techniques were applied, pre-processing, in-processing, post-processing, and their combinations. This procedure resulted in a set of variations, with and without the combined use of the approaches, enabling a comparative analysis of the individual and joint effectiveness of the techniques in bias reduction.

The first approach involved the use of the pre-processing bias mitigation technique known as DEMV [41], with this configuration being referred to as Pre. The second approach consisted of employing an Adversarial Variational Autoencoder (VAE-Adv) with the objective of mitigating bias during model training, characterizing an in-processing technique, designated as In. The third approach employed the post-processing technique MLDebiaser [49], applied to the predictions generated by the classification network, and was identified as Post.

Additionally, combinations of the approaches were evaluated. The joint application of DEMV and VAE-Adv was designated as PI. The combination of DEMV and MLDebiaser was referred to as PP, while the simultaneous use of VAE-Adv and MLDebiaser was identified as IP. Finally, the integrated application of all three approaches, involving pre-processing, in-processing, and post-processing, was designated as PIP.

The optimal hyperparameter values, presented in Table 5, were identified using the Optuna framework with the TPE sampler. These results were derived using the framework applied to the most comprehensive model, PIP, and were subsequently replicated across the other experiments to avoid approach-specific overfitting and to ensure experimental consistency, these optimized hyperparameters were kept fixed and replicated across the remaining experiments.

**Table 5:** The best hyperparameters identified after 2000 epochs of optimization in the Optuna framework

Hyperparameter	DB-PAD-UFES-20	DB-HIBA	DB-MIDAS
lr vae ($lr_{vae}$)	$5.84728e^{-4}$	$5.51749e^{-5}$	$2.46213e^{-4}$
lr adv ($lr_{adv}$)	$1.20033e^{-5}$	$4.16712e^{-3}$	$1.04019e^{-4}$
lambda adv ($\lambda _{adv}$)	0.00322	0.01970	0.08997
beta (*β*)	0.00766	0.29309	0.58789
batch size vae ($b_{size\_vae}$)	48	32	32
optimizer vae ($opt_{vae}$)	Adam	SGD	SGD
batch size mlp ($b_{size\_mlp}$)	16	16	64
optimizer mlp ($opt_{mlp}$)	Adam	Adam	Adam

### DB-PAD-UFES-20

The results related to the performance metrics of the eight models evaluated on the DB-PAD-UFES-20 dataset are listed in Table 6, while the fairness metrics are listed in Table 7. This dataset allows fairness evaluation with respect to two sensitive variables, namely gender and skin phototype according to the Fitzpatrick scale.

For the baseline model, the MLP classifier achieved a BACC of *0.8367 ± 0.0104*. With the application of the pre-processing technique (*Pre*), an improvement in the results was observed, reaching the best overall BACC value, which increased to *0.8414 ± 0.0108*, for the same classifier. For other metrics, the MLP classifier stood out, particularly regarding recall, which reached *0.8647 ± 0.0079* compared to *0.8609 ± 0.0078* in the baseline. In contrast, the behavior of the fairness metrics varied depending on the sensitive variable considered. For gender, Statistical Parity (SP) worsened for the MLP classifier, increasing from *0.0953 ± 0.0094* to *0.1034 ± 0.0176*. For this same variable, the best result was obtained with the Decision Tree (DT) classifier, which reached *0.0825 ± 0.0240*, a value that was still worse than the best result observed in the baseline. For Fitzpatrick, on the other hand, the Equalized Odds Difference (EOD) metric showed considerable improvement with the KNN, achieving the best overall result, dropping from *0.0405 ± 0.0116* to *0.0383 ± 0.0159*.

The in-processing approach (*In*) showed a reduction in performance compared with the baseline when considering the MLP classifier. In contrast, improvements were observed for the KNN and DT classifiers, with the DT achieving a BACC of *0.7581 ± 0.0193* compared with *0.7355 ± 0.0212* for the baseline. However, similarly to the *Pre* approach, the behavior of the fairness metrics varied depending on the sensitive variable considered. For the gender variable, the performance gains were not consistently reflected in the fairness metrics, with improvements observed only for the KNN classifier, while for the others the fairness results were inferior to those of the baseline. For the Fitzpatrick variable, on the other hand, a significant improvement was observed for the DT classifier, which achieved the best overall result for the Disparate Impact (DI) metric, with a value of *0.9944 ± 0.1520*.

The post-processing technique (*Post*) showed a considerable reduction in performance compared to the baseline, achieving a BACC of *0.8184 ± 0.0120* with the MLP. Nevertheless, this approach yielded the best results in the fairness metrics for the gender variable, with an SP of *0.0024 ± 0.0033* and an EOD of *0.0262 ± 0.0103*, both obtained using the MLP. For Fitzpatrick skin type, improvements were observed in the fairness metrics compared with the baseline, although without reaching the best overall results, achieving an SP of *0.0579 ± 0.0036* with the MLP, outperforming the baseline value of *0.1094 ± 0.0101*.

Regarding the combined approaches, the *Pre + In* (PI) configuration achieved the best overall results for ACC (*0.8502 ± 0.0131*), Recall (*0.8502 ± 0.0131*), and F1-Score (*0.8539 ± 0.0121*), all obtained with the MLP classifier, but for BACC the performance remained below the baseline. With respect to the fairness metrics, this approach showed improvements only with the DT classifier, for both the gender and Fitzpatrick variables. Even so, the overall results of this configuration did not stand out consistently in comparison with the other evaluated approaches.

The *Pre + Post* (PP) combination did not outperform the baseline in terms of performance metrics, with the MLP achieving the best result at a BACC of *0.8313 ± 0.0070*. On the other hand, this configuration showed good results in the fairness metrics, achieving an SP and an EOD of *0.0050 ± 0.0063* and *0.0310 ± 0.0136*, respectively, both with the MLP classifier. For Fitzpatrick, it achieved an SP of *0.0580 ± 0.0122* with the MLP, compared with *0.1094 ± 0.0101* in the baseline. The *In + Post* (IP) approach resulted in a slight performance loss, for the MLP classifier and improvements for the KNN and DT classifiers. In the case of the MLP, the BACC reached *0.8202 ± 0.0102*. Regarding the fairness metrics, this configuration showed gains for the gender variable. For the Fitzpatrick variable, the best overall results were obtained for the SP metric, with a value of *0.0274 ± 0.0167* using the KNN classifier, and for the AOD metric, with a value of *0.0919 ± 0.0309* using the MLP classifier.

Finally, the combination of the three approaches (*PIP*) yielded results inferior to the baseline in terms of performance, with the MLP achieving a BACC of *0.8081 ± 0.0116*, the lowest BACC among the MLP models. However, for the fairness metrics related to gender, the results were close to the best overall values. Specifically, the MLP classifier obtained an SP of *0.0054 ± 0.0037* and the best overall result for the AOD metric, with a value of *0.0142 ± 0.0056*, while the DT classifier achieved the best overall result for DI, with a value of *0.9994 ± 0.0658*. For Fitzpatrick, the results outperformed the baseline, although they did not reach the best absolute values.

**Table 6:** Average performance metrics, DB-PAD-UFES-20

Mitigation	Classifier	ACC	BACC	Precision	Recall	F1 Score
None	MLP	*0,8453 ± 0,0094*	*0,8367 ± 0,0104*	*0,8609 ± 0,0078*	*0,8453 ± 0,0094*	*0,8499 ± 0,0088*
	KNN	*0,8057 ± 0,0099*	*0,7455 ± 0,0175*	*0,8044 ± 0,0111*	*0,8057 ± 0,0099*	*0,8048 ± 0,0105*
	DT	*0,8259 ± 0,0068*	*0,7355 ± 0,0212*	*0,8186 ± 0,0075*	*0,8259 ± 0,0068*	*0,8168 ± 0,0101*
Pre	MLP	*0,8494 ± 0,0113*	${\textbf {0,8414}} \pm {\textbf {0,0108}}$	${\textbf {0,8647}} \pm {\textbf {0,0079}}$	*0,8494 ± 0,0113*	*0,8538 ± 0,0103*
	KNN	*0,7992 ± 0,0083*	*0,7352 ± 0,0165*	*0,7973 ± 0,0097*	*0,7992 ± 0,0083*	*0,7978 ± 0,0086*
	DT	*0,8356 ± 0,0137*	*0,7470 ± 0,0424*	*0,8306 ± 0,0171*	*0,8356 ± 0,0137*	*0,8260 ± 0,0197*
In	MLP	*0,8453 ± 0,0065*	*0,8347 ± 0,0061*	*0,8598 ± 0,0048*	*0,8453 ± 0,0065*	*0,8497 ± 0,0059*
	KNN	*0,8089 ± 0,0173*	*0,7507 ± 0,0248*	*0,8080 ± 0,0181*	*0,8089 ± 0,0173*	*0,8082 ± 0,0176*
	DT	*0,8316 ± 0,0098*	*0,7581 ± 0,0193*	*0,8280 ± 0,0078*	*0,8316 ± 0,0098*	*0,8264 ± 0,0087*
PI	MLP	${\textbf {0,8502}} \pm {\textbf {0,0131}}$	*0,8360 ± 0,0134*	*0,8623 ± 0,0100*	${\textbf {0,8502}} \pm {\textbf {0,0131}}$	${\textbf {0,8539}} \pm {\textbf {0,0121}}$
	KNN	*0,8089 ± 0,0047*	*0,7477 ± 0,0132*	*0,8070 ± 0,0068*	*0,8089 ± 0,0047*	*0,8076 ± 0,0056*
	DT	*0,8291 ± 0,0157*	*0,7397 ± 0,0354*	*0,8219 ± 0,0187*	*0,8291 ± 0,0157*	*0,8199 ± 0,0203*
PP	MLP	*0,8308 ± 0,0072*	*0,8313 ± 0,0070*	*0,8550 ± 0,0049*	*0,8308 ± 0,0072*	*0,8371 ± 0,0064*
	KNN	*0,7943 ± 0,0134*	*0,7334 ± 0,0171*	*0,7945 ± 0,0118*	*0,7943 ± 0,0134*	*0,7939 ± 0,0128*
	DT	*0,8385 ± 0,0165*	*0,7776 ± 0,0174*	*0,8361 ± 0,0152*	*0,8385 ± 0,0165*	*0,8358 ± 0,0158*
IP	MLP	*0,8174 ± 0,0071*	*0,8202 ± 0,0102*	*0,8462 ± 0,0073*	*0,8174 ± 0,0071*	*0,8249 ± 0,0068*
	KNN	*0,8174 ± 0,0113*	*0,7624 ± 0,0164*	*0,8170 ± 0,0114*	*0,8174 ± 0,0113*	*0,8169 ± 0,0111*
	DT	*0,8275 ± 0,0224*	*0,7697 ± 0,0286*	*0,8266 ± 0,0216*	*0,8275 ± 0,0224*	*0,8259 ± 0,0215*
Post	MLP	*0,8117 ± 0,0142*	*0,8184 ± 0,0120*	*0,8447 ± 0,0091*	*0,8117 ± 0,0142*	*0,8199 ± 0,0130*
	KNN	*0,8012 ± 0,0158*	*0,7449 ± 0,0181*	*0,8025 ± 0,0135*	*0,8012 ± 0,0158*	*0,8014 ± 0,0151*
	DT	*0,8409 ± 0,0098*	*0,7798 ± 0,0175*	*0,8374 ± 0,0105*	*0,8409 ± 0,0098*	*0,8381 ± 0,0105*
PIP	MLP	*0,8061 ± 0,0104*	*0,8081 ± 0,0116*	*0,8372 ± 0,0086*	*0,8061 ± 0,0104*	*0,8142 ± 0,0097*
	KNN	*0,8073 ± 0,0152*	*0,7515 ± 0,0121*	*0,8086 ± 0,0113*	*0,8073 ± 0,0152*	*0,8072 ± 0,0131*
	DT	*0,8223 ± 0,0273*	*0,7657 ± 0,0337*	*0,8214 ± 0,0270*	*0,8223 ± 0,0273*	*0,8211 ± 0,0272*

**Table 7:** Average fairness metrics, DB-PAD-UFES-20

Mitigation	Classifier	Statistical Parity	Disparate Impact	Equal Opportunity Diff	Average Odds Diff
Gender					
None	MLP	*0,0953 ± 0,0094*	*1,1515 ± 0,0163*	*0,0832 ± 0,0106*	*0,0464 ± 0,0089*
	KNN	*0,1245 ± 0,0349*	*1,1843 ± 0,0561*	*0,1081 ± 0,0342*	*0,0960 ± 0,0473*
	DT	*0,0728 ± 0,0183*	*1,0956 ± 0,0252*	*0,0527 ± 0,0130*	*0,0492 ± 0,0270*
Pre	MLP	*0,1034 ± 0,0176*	*1,1664 ± 0,0352*	*0,0984 ± 0,0159*	*0,0473 ± 0,0192*
	KNN	*0,1179 ± 0,0295*	*1,1727 ± 0,0483*	*0,1171 ± 0,0269*	*0,0771 ± 0,0425*
	DT	*0,0825 ± 0,0240*	*1,1107 ± 0,0404*	*0,0631 ± 0,0043*	*0,0563 ± 0,0423*
In	MLP	*0,1114 ± 0,0128*	*1,1784 ± 0,0208*	*0,0963 ± 0,0105*	*0,0657 ± 0,0152*
	KNN	*0,1180 ± 0,0234*	*1,1732 ± 0,0345*	*0,0882 ± 0,0330*	*0,1008 ± 0,0300*
	DT	*0,0945 ± 0,0437*	*1,1341 ± 0,0716*	*0,0671 ± 0,0268*	*0,0736 ± 0,0597*
PI	MLP	*0,1145 ± 0,0208*	*1,1821 ± 0,0369*	*0,0999 ± 0,0205*	*0,0680 ± 0,0238*
	KNN	*0,0985 ± 0,0195*	*1,1416 ± 0,0296*	*0,0857 ± 0,0126*	*0,0662 ± 0,0338*
	DT	*0,0728 ± 0,0287*	*1,0967 ± 0,0420*	*0,0437 ± 0,0392*	*0,0566 ± 0,0282*
PP	MLP	*0,0050 ± 0,0063*	*0,9931 ± 0,0098*	*0,0310 ± 0,0136*	*0,0389 ± 0,0143*
	KNN	*0,0876 ± 0,0450*	*1,1139 ± 0,0915*	*0,0826 ± 0,0362*	*0,0665 ± 0,0500*
	DT	*0,0511 ± 0,0347*	*1,0040 ± 0,0816*	*0,0527 ± 0,0295*	*0,0699 ± 0,0628*
IP	MLP	*0,0033 ± 0,0035*	*0,9958 ± 0,0061*	*0,0362 ± 0,0108*	*0,0286 ± 0,0126*
	KNN	*0,1181 ± 0,0172*	*1,1738 ± 0,0273*	*0,0922 ± 0,0147*	*0,0952 ± 0,0257*
	DT	*0,0461 ± 0,0275*	*1,0266 ± 0,0673*	*0,0377 ± 0,0188*	*0,0474 ± 0,0324*
Post	MLP	${\textbf {0,0024}} \pm {\textbf {0,0033}}$	*1,0038 ± 0,0054*	${\textbf {0,0262}} \pm {\textbf {0,0103}}$	*0,0269 ± 0,0066*
	KNN	*0,1007 ± 0,0508*	*1,1355 ± 0,1016*	*0,0847 ± 0,0417*	*0,0876 ± 0,0487*
	DT	*0,0350 ± 0,0255*	*1,0364 ± 0,0459*	*0,0303 ± 0,0239*	*0,0440 ± 0,0267*
PIP	MLP	*0,0054 ± 0,0037*	*1,0036 ± 0,0095*	*0,0398 ± 0,0197*	${\textbf {0,0142}} \pm {\textbf {0,0056}}$
	KNN	*0,0957 ± 0,0507*	*1,1414 ± 0,0790*	*0,0753 ± 0,0335*	*0,0833 ± 0,0640*
	DT	*0,0458 ± 0,0209*	${\textbf {0,9994}} \pm {\textbf {0,0658}}$	*0,0453 ± 0,0097*	*0,0518 ± 0,0401*
Fitzpatrick					
None	MLP	*0,1094 ± 0,0101*	*0,8395 ± 0,0124*	*0,1947 ± 0,0117*	*0,1895 ± 0,0111*
	KNN	*0,0299 ± 0,0159*	*0,9603 ± 0,0205*	*0,0405 ± 0,0116*	*0,2140 ± 0,0225*
	DT	*0,1789 ± 0,0719*	*0,7789 ± 0,0883*	*0,1996 ± 0,0815*	*0,3311 ± 0,0508*
Pre	MLP	*0,1102 ± 0,0168*	*0,8388 ± 0,0212*	*0,1981 ± 0,0178*	*0,1881 ± 0,0176*
	KNN	*0,0339 ± 0,0204*	*0,9564 ± 0,0280*	${\textbf {0,0383}} \pm {\textbf {0,0159}}$	*0,2223 ± 0,0279*
	DT	*0,1218 ± 0,0798*	*0,8528 ± 0,0954*	*0,1386 ± 0,0745*	*0,2928 ± 0,0782*
In	MLP	*0,1127 ± 0,0085*	*0,8353 ± 0,0103*	*0,1970 ± 0,0095*	*0,1938 ± 0,0086*
	KNN	*0,0576 ± 0,0560*	*0,9225 ± 0,0760*	*0,0761 ± 0,0759*	*0,2271 ± 0,0277*
	DT	*0,0944 ± 0,0743*	${\textbf {0,9944}} \pm {\textbf {0,1520}}$	*0,0777 ± 0,0302*	*0,2638 ± 0,0637*
PI	MLP	*0,1211 ± 0,0180*	*0,8257 ± 0,0214*	*0,2061 ± 0,0192*	*0,2015 ± 0,0192*
	KNN	*0,0332 ± 0,0176*	*0,9561 ± 0,0229*	*0,0451 ± 0,0117*	*0,2163 ± 0,0233*
	DT	*0,0986 ± 0,0809*	*0,8878 ± 0,1086*	*0,1258 ± 0,0771*	*0,2774 ± 0,0583*
PP	MLP	*0,0580 ± 0,0122*	*1,0476 ± 0,0779*	*0,0417 ± 0,0551*	*0,0967 ± 0,0270*
	KNN	*0,0340 ± 0,0159*	*0,9549 ± 0,0205*	*0,0383 ± 0,0105*	*0,2238 ± 0,0227*
	DT	*0,1095 ± 0,0635*	*0,8571 ± 0,0851*	*0,1515 ± 0,0700*	*0,2508 ± 0,0498*
IP	MLP	*0,0680 ± 0,0054*	*1,0641 ± 0,0844*	*0,0402 ± 0,0530*	${\textbf {0,0919}} \pm {\textbf {0,0309}}$
	KNN	${\textbf {0,0274}} \pm {\textbf {0,0167}}$	*0,9636 ± 0,0215*	*0,0473 ± 0,0148*	*0,2034 ± 0,0215*
	DT	*0,0652 ± 0,0553*	*0,9444 ± 0,0999*	*0,0928 ± 0,0731*	*0,2125 ± 0,0352*
Post	MLP	*0,0579 ± 0,0036*	*0,9829 ± 0,0891*	*0,0841 ± 0,0595*	*0,1183 ± 0,0322*
	KNN	*0,0299 ± 0,0159*	*0,9603 ± 0,0205*	*0,0405 ± 0,0116*	*0,2140 ± 0,0225*
	DT	*0,0801 ± 0,0452*	*0,8946 ± 0,0623*	*0,1159 ± 0,0635*	*0,2345 ± 0,0248*
PIP	MLP	*0,0645 ± 0,0030*	*0,9786 ± 0,0984*	*0,0890 ± 0,0585*	*0,1295 ± 0,0337*
	KNN	*0,0315 ± 0,0151*	*0,9581 ± 0,0190*	*0,0473 ± 0,0169*	*0,2112 ± 0,0167*
	DT	*0,0492 ± 0,0663*	*0,9424 ± 0,0938*	*0,1106 ± 0,0903*	*0,2124 ± 0,0462*

### DB-HIBA

The performance metric results for the eight evaluated approaches are presented in Table 8, while the fairness metrics are listed in Table 9. The model that does not incorporate any bias mitigation technique is used as the baseline and is denoted as None. This configuration achieved the best overall performance among the evaluated approaches. In particular, the MLP classifier reached a balanced accuracy (BACC) of *0.8390 ± 0.0081*, however, with the worst values in the fairness metrics.

The results obtained with the DEMV pre-processing technique are presented in Table 8 under the label *Pre*. The data indicated a slight decrease in the performance of the MLP classifier, with a Balanced Accuracy (BACC) of *0.8352 ± 0.0075*, compared to the best baseline result, also obtained by the MLP (*0.8390 ± 0.0081*). Furthermore, a reduction in fairness metric values was observed, with the results reported in Table 9, most notably regarding Statistical Parity (SP), which decreased from *0.1284 ± 0.0152* to *0.1131 ± 0.0162* for the KNN classifier, with other metrics exhibiting similar behavior.

In the in-processing approach (*In*), a slight reduction in performance was observed compared to the baseline, accompanied by improvements in the fairness metrics. However, in comparison with the *Pre* approach, superiority was verified in some metrics. Specifically, for the KNN classifier, the Equal Opportunity Difference (EOD) reached *0.0728 ± 0.0609*, in contrast to *0.1050 ± 0.0322*, which corresponded to the best result previously obtained by the *Pre* approach with the KNN classifier.

The post-processing approach (*Post*) showed a considerable reduction in performance in the BACC metric for the MLP classifier, which decreased from *0.8390 ± 0.0081* to *0.7922 ± 0.0070*. However, this approach achieved the best overall results in Disparate Impact (DI) fairness metric, reaching *1.0029 ± 0.0154*, in contrast to *1.4277 ± 0.0794* obtained by the baseline model with the same classifier.

Regarding the combinations, the *Pre + In* (PI) aggregation resulted in a slight reduction in performance for the MLP classifier; however, it showed a considerable improvement across all fairness metrics, particularly for the Equalized Odds Difference (EOD), which reached *0.0472 ± 0.0448* with the KNN classifier. Conversely, the *Pre + Post* (PP) combination did not produce performance gains but achieved the best overall result with the Decision Tree (DT) classifier for the EOD metric, with a value of *0.0378 ± 0.0341*, and for the Average Odds Difference (AOD), with a value of *0.0380 ± 0.0245*.

The *In + Post* (IP) approach did not outperform the baseline model in performance metrics. Nevertheless, it achieved significant reductions in fairness metrics when associated with the MLP and DT classifiers, although it did not stand out as the best overall strategy. Finally, the combination of the three approaches, *Pre + In + Post* (PIP), did not yield performance gains but achieved the best result in the SP metric, with a value of *0.0046 ± 0.0050*, and the second-best result for the AOD metric, with a value of *0.0389 ± 0.0274* when using the KNN classifier, highlighting the trade-off between performance and fairness.

**Table 8:** Average performance metrics, DB-HIBA

Mitigation	Classifier	ACC	BACC	Precision	Recall	F1 Score
None	MLP	${\textbf {0,8386}} \pm {\textbf {0,0087}}$	${\textbf {0,8390}} \pm {\textbf {0,0081}}$	${\textbf {0,8407}} \pm {\textbf {0,0081}}$	${\textbf {0,8386}} \pm {\textbf {0,0087}}$	${\textbf {0,8390}} \pm {\textbf {0,0086}}$
	KNN	*0,8000 ± 0,0135*	*0,7898 ± 0,0139*	*0,8001 ± 0,0138*	*0,8000 ± 0,0135*	*0,7982 ± 0,0136*
	DT	*0,7868 ± 0,0056*	*0,7768 ± 0,0090*	*0,7870 ± 0,0052*	*0,7868 ± 0,0056*	*0,7849 ± 0,0067*
Pre	MLP	*0,8345 ± 0,0082*	*0,8352 ± 0,0075*	*0,8369 ± 0,0074*	*0,8345 ± 0,0082*	*0,8350 ± 0,0081*
	KNN	*0,8010 ± 0,0067*	*0,7901 ± 0,0070*	*0,8015 ± 0,0070*	*0,8010 ± 0,0067*	*0,7990 ± 0,0068*
	DT	*0,7878 ± 0,0138*	*0,7785 ± 0,0153*	*0,7876 ± 0,0139*	*0,7878 ± 0,0138*	*0,7863 ± 0,0142*
In	MLP	*0,8213 ± 0,0087*	*0,8233 ± 0,0113*	*0,8257 ± 0,0111*	*0,8213 ± 0,0087*	*0,8219 ± 0,0089*
	KNN	*0,7898 ± 0,0169*	*0,7797 ± 0,0205*	*0,7901 ± 0,0162*	*0,7898 ± 0,0169*	*0,7879 ± 0,0182*
	DT	*0,8081 ± 0,0093*	*0,8037 ± 0,0108*	*0,8096 ± 0,0098*	*0,8081 ± 0,0093*	*0,8077 ± 0,0093*
PI	MLP	*0,8203 ± 0,0105*	*0,8235 ± 0,0109*	*0,8264 ± 0,0109*	*0,8203 ± 0,0105*	*0,8210 ± 0,0104*
	KNN	*0,8010 ± 0,0108*	*0,7921 ± 0,0115*	*0,8009 ± 0,0108*	*0,8010 ± 0,0108*	*0,7997 ± 0,0111*
	DT	*0,7919 ± 0,0096*	*0,7883 ± 0,0138*	*0,7942 ± 0,0129*	*0,7919 ± 0,0096*	*0,7915 ± 0,0100*
PP	MLP	*0,7929 ± 0,0122*	*0,7886 ± 0,0143*	*0,7930 ± 0,0130*	*0,7929 ± 0,0122*	*0,7928 ± 0,0125*
	KNN	*0,7949 ± 0,0149*	*0,7848 ± 0,0166*	*0,7950 ± 0,0146*	*0,7949 ± 0,0149*	*0,7931 ± 0,0155*
	DT	*0,7665 ± 0,0151*	*0,7572 ± 0,0178*	*0,7661 ± 0,0155*	*0,7665 ± 0,0151*	*0,7649 ± 0,0158*
IP	MLP	*0,7827 ± 0,0141*	*0,7803 ± 0,0141*	*0,7838 ± 0,0139*	*0,7827 ± 0,0141*	*0,7830 ± 0,0140*
	KNN	*0,8000 ± 0,0192*	*0,7918 ± 0,0201*	*0,8002 ± 0,0192*	*0,8000 ± 0,0192*	*0,7988 ± 0,0193*
	DT	*0,7980 ± 0,0171*	*0,7891 ± 0,0209*	*0,7985 ± 0,0165*	*0,7980 ± 0,0171*	*0,7964 ± 0,0183*
Post	MLP	*0,7959 ± 0,0038*	*0,7922 ± 0,0038*	*0,7960 ± 0,0038*	*0,7959 ± 0,0038*	*0,7960 ± 0,0038*
	KNN	*0,8000 ± 0,0135*	*0,7898 ± 0,0139*	*0,8001 ± 0,0138*	*0,8000 ± 0,0135*	*0,7982 ± 0,0136*
	DT	*0,7716 ± 0,0161*	*0,7645 ± 0,0148*	*0,7711 ± 0,0159*	*0,7716 ± 0,0161*	*0,7708 ± 0,0157*
PIP	MLP	*0,7766 ± 0,0072*	*0,7744 ± 0,0070*	*0,7780 ± 0,0070*	*0,7766 ± 0,0072*	*0,7770 ± 0,0071*
	KNN	*0,7827 ± 0,0168*	*0,7743 ± 0,0174*	*0,7822 ± 0,0169*	*0,7827 ± 0,0168*	*0,7815 ± 0,0169*
	DT	*0,7756 ± 0,0113*	*0,7669 ± 0,0111*	*0,7753 ± 0,0110*	*0,7756 ± 0,0113*	*0,7743 ± 0,0112*

**Table 9:** Average fairness metrics, DB-HIBA

Mitigation	Classifier	Statistical Parity	Disparate Impact	Equal Opportunity Diff	Average Odds Diff
Gender					
None	MLP	*0,1627 ± 0,0247*	*1,4277 ± 0,0794*	*0,1367 ± 0,0392*	*0,0790 ± 0,0252*
	KNN	*0,1284 ± 0,0152*	*1,3944 ± 0,0630*	*0,1328 ± 0,0292*	*0,0594 ± 0,0193*
	DT	*0,1597 ± 0,0155*	*1,5043 ± 0,0373*	*0,1861 ± 0,0288*	*0,0968 ± 0,0154*
Pre	MLP	*0,1590 ± 0,0239*	*1,4149 ± 0,0833*	*0,1228 ± 0,0355*	*0,0752 ± 0,0241*
	KNN	*0,1131 ± 0,0162*	*1,3446 ± 0,0577*	*0,1050 ± 0,0322*	*0,0425 ± 0,0164*
	DT	*0,1628 ± 0,0208*	*1,5074 ± 0,0568*	*0,1900 ± 0,0323*	*0,0995 ± 0,0189*
In	MLP	*0,1592 ± 0,0326*	*1,4085 ± 0,1065*	*0,0933 ± 0,0577*	*0,0748 ± 0,0364*
	KNN	*0,1124 ± 0,0402*	*1,3418 ± 0,1440*	*0,0728 ± 0,0609*	*0,0427 ± 0,0381*
	DT	*0,1568 ± 0,0247*	*1,4499 ± 0,1054*	*0,1167 ± 0,0770*	*0,0803 ± 0,0287*
PI	MLP	*0,1384 ± 0,0242*	*1,3387 ± 0,0851*	*0,0794 ± 0,0338*	*0,0547 ± 0,0231*
	KNN	*0,1240 ± 0,0358*	*1,3730 ± 0,1309*	*0,0472 ± 0,0448*	*0,0454 ± 0,0380*
	DT	*0,1215 ± 0,0270*	*1,3279 ± 0,0909*	*0,0894 ± 0,0400*	*0,0490 ± 0,0287*
PP	MLP	*0,0102 ± 0,0033*	*0,9893 ± 0,0227*	*0,0894 ± 0,0135*	*0,0847 ± 0,0078*
	KNN	*0,1102 ± 0,0334*	*1,3363 ± 0,1233*	*0,1144 ± 0,0517*	*0,0442 ± 0,0367*
	DT	*0,0354 ± 0,0306*	*1,0915 ± 0,0821*	${\textbf {0,0378}} \pm {\textbf {0,0341}}$	${\textbf {0,0380}} \pm {\textbf {0,0245}}$
IP	MLP	*0,0073 ± 0,0031*	*1,0054 ± 0,0173*	*0,1278 ± 0,0292*	*0,0803 ± 0,0075*
	KNN	*0,1558 ± 0,0299*	*1,4849 ± 0,1351*	*0,1167 ± 0,0338*	*0,0823 ± 0,0321*
	DT	*0,0787 ± 0,0703*	*1,1849 ± 0,2395*	*0,1161 ± 0,0450*	*0,0824 ± 0,0202*
Post	MLP	*0,0047 ± 0,0042*	${\textbf {1,0053}} \pm {\textbf {0,0138}}$	*0,0856 ± 0,0177*	*0,0790 ± 0,0069*
	KNN	*0,1284 ± 0,0152*	*1,3944 ± 0,0630*	*0,1328 ± 0,0292*	*0,0594 ± 0,0193*
	DT	*0,0285 ± 0,0351*	*1,0537 ± 0,1152*	*0,0672 ± 0,0132*	*0,0595 ± 0,0242*
PIP	MLP	${\textbf {0,0046}} \pm {\textbf {0,0050}}$	*1,0064 ± 0,0142*	*0,1178 ± 0,0237*	*0,0775 ± 0,0027*
	KNN	*0,1088 ± 0,0309*	*1,3095 ± 0,0896*	*0,0439 ± 0,0302*	*0,0389 ± 0,0274*
	DT	*0,0730 ± 0,0367*	*0,8634 ± 0,1172*	*0,2106 ± 0,0633*	*0,1439 ± 0,0531*

### DB-MIDAS

Applying the same experimental setup to the DB-MIDAS dataset, we present the performance metrics for the eight evaluated approaches in Table 10, whereas the fairness metrics are listed in Table 11.

Consistent with the previous experiment, the models without the application of any mitigation techniques presented the best overall performance results. Specifically, the KNN classifier achieved a BACC of *0.6987 ± 0.0102*, but exhibited the worst values in the fairness metrics.

In the pre-processing approach (*Pre*), the best performance was achieved with the KNN classifier, with a BACC of *0.6946 ± 0.0076*, which is lower than the baseline. In terms of fairness, this approach led to an improvement in SP, which was reduced from *0.0995 ± 0.0224* to *0.0894 ± 0.0152*, and in DI, which decreased from *1.2109 ± 0.0416* to *1.1862 ± 0.0187* for the DT classifier. Additionally, this strategy achieved the best overall result for the AOD metric, reaching *0.0312 ± 0.0191*.

Regarding the in-processing approach (*In*), an inverse behavior was observed among the classifiers, the MLP achieved the best performance in terms of BACC, however, it obtained the worst results in fairness. In contrast, the DT yielded the best fairness metrics, but showed the lowest performance among the classifiers.

With the post-processing approach (*Post*), the best performance was achieved by the KNN, reaching a BACC of *0.6987 ± 0.0102*, similar to the best overall model. However, gains in fairness metrics were observed, most notably for SP, which reached the best overall value (*0.0069 ± 0.0037*) with the MLP classifier, in addition to improvements in the other fairness metrics compared with the baseline, except for EOD, which showed a decline. The *Pre* + *In* (PI) combination resulted in significant performance losses, with the best observed result being *0.6791 ± 0.0234*, lower than the baseline. Despite this, some classifiers achieved fairness metrics that were better than those of the baseline.

In the *Pre + Post* (PP) configuration, the KNN classifier achieved the best performance, with a BACC of *0.6986 ± 0.0069*. Regarding the fairness metrics, the MLP classifier obtained the second-best result for the SP metric, with *0.0096 ± 0.0081*. For the *In + Post* (IP) approach, the best results were obtained with the MLP, which presented a BACC of *0.6719 ± 0.0266* and reached the lowest overall value for the EOD metric (*0.0352 ± 0.0339*). In addition, the best overall result for the DI metric was obtained with the DT classifier, reaching *0.9993 ± 0.1823*. Finally, the combination of the three techniques, *Pre + In + Post* (PIP), showed a decrease in performance values, with the highest being recorded by the MLP at a BACC of only *0.6561 ± 0.0153*. However, this configuration still presented fairness results superior to those of the baseline.

**Table 10:** Average performance metrics, DB-MIDAS

Mitigation	Classifier	ACC	BACC	Precision	Recall	F1 Score
None	MLP	*0,6882 ± 0,0107*	*0,6887 ± 0,0103*	*0,6896 ± 0,0096*	*0,6882 ± 0,0107*	*0,6880 ± 0,0110*
	KNN	${\textbf {0,7000}} \pm {\textbf {0,0098}}$	${\textbf {0,6987}} \pm {\textbf {0,0102}}$	${\textbf {0,7012}} \pm {\textbf {0,0092}}$	${\textbf {0,7000}} \pm {\textbf {0,0098}}$	${\textbf {0,6989}} \pm {\textbf {0,0104}}$
	DT	*0,6947 ± 0,0122*	*0,6939 ± 0,0122*	*0,6964 ± 0,0119*	*0,6947 ± 0,0122*	*0,6937 ± 0,0125*
Pre	MLP	*0,6895 ± 0,0158*	*0,6900 ± 0,0159*	*0,6911 ± 0,0157*	*0,6895 ± 0,0158*	*0,6892 ± 0,0160*
	KNN	*0,6961 ± 0,0077*	*0,6946 ± 0,0076*	*0,6976 ± 0,0082*	*0,6961 ± 0,0077*	*0,6948 ± 0,0076*
	DT	*0,6908 ± 0,0072*	*0,6899 ± 0,0071*	*0,6928 ± 0,0084*	*0,6908 ± 0,0072*	*0,6896 ± 0,0069*
In	MLP	*0,6776 ± 0,0166*	*0,6781 ± 0,0160*	*0,6815 ± 0,0151*	*0,6776 ± 0,0166*	*0,6763 ± 0,0173*
	KNN	*0,6263 ± 0,0240*	*0,6249 ± 0,0247*	*0,6276 ± 0,0225*	*0,6263 ± 0,0240*	*0,6237 ± 0,0269*
	DT	*0,5776 ± 0,0375*	*0,5754 ± 0,0385*	*0,5774 ± 0,0382*	*0,5776 ± 0,0375*	*0,5737 ± 0,0400*
PI	MLP	*0,6789 ± 0,0240*	*0,6791 ± 0,0234*	*0,6802 ± 0,0230*	*0,6789 ± 0,0240*	*0,6786 ± 0,0242*
	KNN	*0,6526 ± 0,0113*	*0,6513 ± 0,0128*	*0,6551 ± 0,0100*	*0,6526 ± 0,0113*	*0,6502 ± 0,0135*
	DT	*0,5921 ± 0,0335*	*0,5897 ± 0,0305*	*0,6017 ± 0,0401*	*0,5921 ± 0,0335*	*0,5823 ± 0,0292*
PP	MLP	*0,6908 ± 0,0059*	*0,6911 ± 0,0056*	*0,6916 ± 0,0055*	*0,6908 ± 0,0059*	*0,6907 ± 0,0059*
	KNN	*0,7000 ± 0,0067*	*0,6986 ± 0,0069*	*0,7015 ± 0,0066*	*0,7000 ± 0,0067*	*0,6988 ± 0,0070*
	DT	*0,6684 ± 0,0255*	*0,6678 ± 0,0249*	*0,6689 ± 0,0254*	*0,6684 ± 0,0255*	*0,6680 ± 0,0252*
IP	MLP	*0,6711 ± 0,0279*	*0,6719 ± 0,0266*	*0,6755 ± 0,0254*	*0,6711 ± 0,0279*	*0,6695 ± 0,0288*
	KNN	*0,6513 ± 0,0216*	*0,6502 ± 0,0226*	*0,6524 ± 0,0212*	*0,6513 ± 0,0216*	*0,6498 ± 0,0228*
	DT	*0,5829 ± 0,0219*	*0,5801 ± 0,0224*	*0,5858 ± 0,0214*	*0,5829 ± 0,0219*	*0,5751 ± 0,0255*
Post	MLP	*0,6921 ± 0,0147*	*0,6925 ± 0,0143*	*0,6932 ± 0,0140*	*0,6921 ± 0,0147*	*0,6920 ± 0,0148*
	KNN	*0,7000 ± 0,0098*	*0,6987 ± 0,0102*	*0,7012 ± 0,0092*	*0,7000 ± 0,0098*	*0,6989 ± 0,0104*
	DT	*0,6711 ± 0,0367*	*0,6712 ± 0,0361*	*0,6723 ± 0,0362*	*0,6711 ± 0,0367*	*0,6707 ± 0,0367*
PIP	MLP	*0,6553 ± 0,0159*	*0,6561 ± 0,0153*	*0,6583 ± 0,0141*	*0,6553 ± 0,0159*	*0,6543 ± 0,0169*
	KNN	*0,6184 ± 0,0161*	*0,6180 ± 0,0164*	*0,6188 ± 0,0163*	*0,6184 ± 0,0161*	*0,6180 ± 0,0163*
	DT	*0,5947 ± 0,0464*	*0,5935 ± 0,0462*	*0,5985 ± 0,0454*	*0,5947 ± 0,0464*	*0,5891 ± 0,0485*

**Table 11:** Average fairness metrics, DB-MIDAS

Mitigation	Classifier	Statistical Parity	Disparate Impact	Equal Opportunity Diff	Average Odds Diff
Gender					
None	MLP	*0,1322 ± 0,0324*	*1,3390 ± 0,0947*	*0,0528 ± 0,0410*	*0,0736 ± 0,0324*
	KNN	*0,1328 ± 0,0390*	*1,2881 ± 0,0878*	*0,1731 ± 0,0410*	*0,0806 ± 0,0341*
	DT	*0,0995 ± 0,0224*	*1,2109 ± 0,0416*	*0,0662 ± 0,0237*	*0,0398 ± 0,0244*
Pre	MLP	*0,1423 ± 0,0289*	*1,3744 ± 0,1020*	*0,0569 ± 0,0607*	*0,0827 ± 0,0305*
	KNN	*0,1425 ± 0,0419*	*1,3095 ± 0,0949*	*0,1766 ± 0,0360*	*0,0912 ± 0,0368*
	DT	*0,0894 ± 0,0152*	*1,1862 ± 0,0187*	*0,0628 ± 0,0387*	${\textbf {0,0312}} \pm {\textbf {0,0191}}$
In	MLP	*0,1120 ± 0,0361*	*1,2951 ± 0,1495*	*0,0866 ± 0,0552*	*0,0589 ± 0,0344*
	KNN	*0,0552 ± 0,0613*	*1,1220 ± 0,1553*	*0,0717 ± 0,0720*	*0,0626 ± 0,0348*
	DT	*0,0395 ± 0,0273*	*1,0719 ± 0,0510*	*0,1403 ± 0,0407*	*0,0331 ± 0,0208*
PI	MLP	*0,1355 ± 0,0305*	*1,3333 ± 0,0804*	*0,1134 ± 0,0561*	*0,0842 ± 0,0283*
	KNN	*0,1274 ± 0,0419*	*1,2250 ± 0,2069*	*0,1783 ± 0,0952*	*0,1054 ± 0,0393*
	DT	*0,0566 ± 0,0422*	*1,0403 ± 0,1151*	*0,0845 ± 0,0560*	*0,0554 ± 0,0186*
PP	MLP	*0,0096 ± 0,0081*	*1,0117 ± 0,0239*	*0,0769 ± 0,0315*	*0,0560 ± 0,0157*
	KNN	*0,1366 ± 0,0425*	*1,2966 ± 0,0952*	*0,1766 ± 0,0360*	*0,0845 ± 0,0372*
	DT	*0,0737 ± 0,0253*	*1,0418 ± 0,1459*	*0,0603 ± 0,0338*	*0,0641 ± 0,0439*
IP	MLP	*0,0101 ± 0,0066*	*0,9900 ± 0,0262*	${\textbf {0,0352}} \pm {\textbf {0,0339}}$	*0,0558 ± 0,0115*
	KNN	*0,0951 ± 0,0673*	*1,2225 ± 0,1818*	*0,1510 ± 0,0873*	*0,0601 ± 0,0585*
	DT	*0,0771 ± 0,0593*	${\textbf {0,9993}} \pm {\textbf {0,1823}}$	*0,0828 ± 0,0959*	*0,0867 ± 0,0607*
Post	MLP	${\textbf {0,0069}} \pm {\textbf {0,0037}}$	*1,0111 ± 0,0111*	*0,0848 ± 0,0532*	*0,0576 ± 0,0035*
	KNN	*0,1328 ± 0,0390*	*1,2881 ± 0,0878*	*0,1731 ± 0,0410*	*0,0806 ± 0,0341*
	DT	*0,0819 ± 0,0232*	*1,0037 ± 0,1717*	*0,0917 ± 0,0410*	*0,0845 ± 0,0435*
PIP	MLP	*0,0152 ± 0,0095*	*0,9910 ± 0,0386*	*0,0724 ± 0,0491*	*0,0528 ± 0,0199*
	KNN	*0,0724 ± 0,0284*	*1,1549 ± 0,0621*	*0,1121 ± 0,0752*	*0,0499 ± 0,0168*
	DT	*0,0529 ± 0,0260*	*0,9954 ± 0,1194*	*0,0821 ± 0,0675*	*0,0453 ± 0,0326*

### Discussion

For the DB-PAD-UFES-20 dataset, the best performance results were obtained using the Pre-processing (*Pre*) mitigation technique in conjunction with the MLP classifier. It achieved a BACC of *0.8414 ± 0.0108*, representing a *0.56%* improvement over the baseline. The remaining performance metrics showed the best values with the *PI* mitigation technique.

The analysis of the fairness metrics was conducted separately for the sensitive variables gender and Fitzpatrick. For the gender variable, the post-processing technique (Post) with the MLP classifier achieved a *96,7%* percentage improvement in Statistical Parity (SP) and an improvement of *50.3%* in the Equal Opportunity Difference (EOD) metric compared with the best baseline result (DT). For Disparate Impact (DI), the best configuration was the *Pre + In + Post* (PIP) combination using the same classifier (MLP), showing an improvement of *13.21%* relative to the MLP baseline and *8.78%* over the DT baseline. For Average Odds Difference (AOD), the best result was also obtained with the PIP combination, in which the MLP achieved a *69.4%* gain.

For the Fitzpatrick variable, the SP metric achieved its best result with the *In + Post* (IP) approach using the KNN classifier, reaching an improvement of *8.36%*. This same combination also produced the best result for AOD with the MLP classifier, with an increase of *51.50%*. DI showed a significant improvement with the *In* approach, achieving a gain of *27.67%* with DT classifier and reaching values close to the ideal. Finally, the *Pre* approach with the KNN classifier achieved the best performance for the EOD metric, with an improvement of *5.43%* compared with the baseline.

With the aim of identifying the best combination of mitigation approach and classifier for each dataset, a global evaluation was performed considering all metrics, using the similarity of the results to an ideal solution. For this purpose, the A-TOPSIS algorithm [32] was applied, which allows for the ranking of algorithms based on the mean values and standard deviations of the evaluated metrics.

In the ranking process, the performance metrics and DI were defined as benefit criteria, where the optimal solution is given by a value of 1.0. On the other hand, the remaining fairness metrics were treated as cost criteria, with an ideal solution represented by a value of 0.0. All criteria were assigned equal weights, and the data were previously normalized to ensure comparability across metrics.

**Fig. 6:**
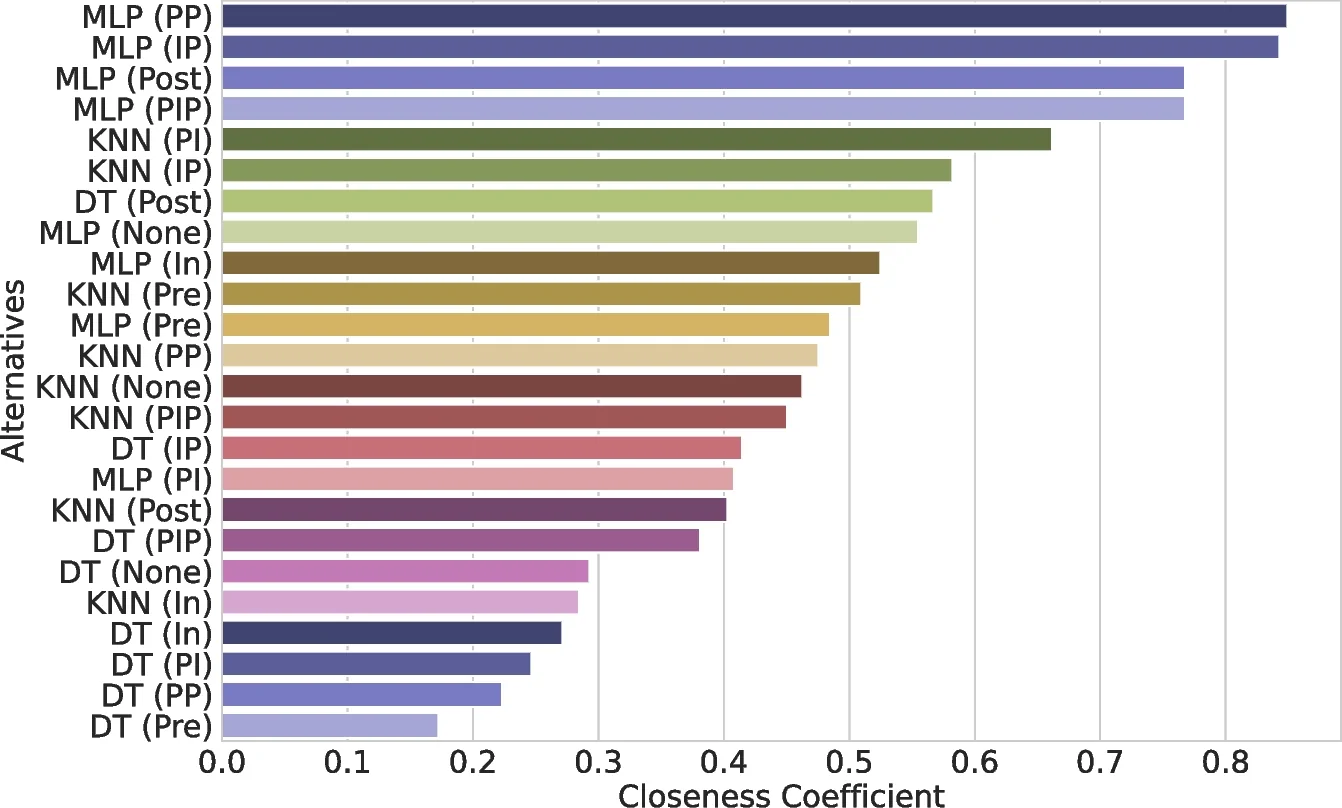
Visualization of the A-TOPSIS rankings coefficient for the DB-PAD-UFES-20 dataset

When applying A-TOPSIS to derive the rankings, it was observed that the three highest-ranked configurations used the MLP classifier. The first position was assigned to the *Pre + Post* (PP) approach, followed by the *In + Post* (IP) approach, while the *Post* approach occupied the third position. These results are depicted in Fig. 6.

These results highlight the trade-off between performance metrics and fairness metrics. Although the *Pre* approach with MLP achieved the best performance values, its application led to a deterioration in fairness metrics when compared to the baseline. The global analysis, however, identifies the *PP* approach with MLP as the most balanced configuration, incurring only a *1.20%* loss in BACC relative to the best performance and therefore being considered the best alternative for this dataset based on the adopted criteria. Although it did not achieve the best absolute values in the fairness metrics, the ranking obtained by the A-TOPSIS indicates that this approach provides the best balance between performance and fairness.

For the DB-HIBA dataset, the best performance results were achieved by the model without applying mitigation techniques (*None*) with the MLP classifier, reaching a balanced accuracy (BACC) of *0.8390 ± 0.0081*. Regarding fairness metrics, no single approach was identified that simultaneously achieved the best results across all four evaluated metrics.

The standalone post-processing technique yielded the best results for DI, with a percentage improvement of *29.59%* compared with the baseline using the MLP classifier. On the other hand, the combination of the three mitigation techniques (*PIP*) obtained the best result for the SP metric, showing an improvement of *97.17%* over the best baseline model. For the EOD and AOD metrics, the best results were achieved by the *Pre + Post* (PP) combination with the DT classifier, corresponding to improvements of *79.69%* and *60.74%*, respectively, compared with the baseline. These results indicate that, for this type of data, there is no single approach capable of optimizing all fairness metrics simultaneously.

**Fig. 7:**
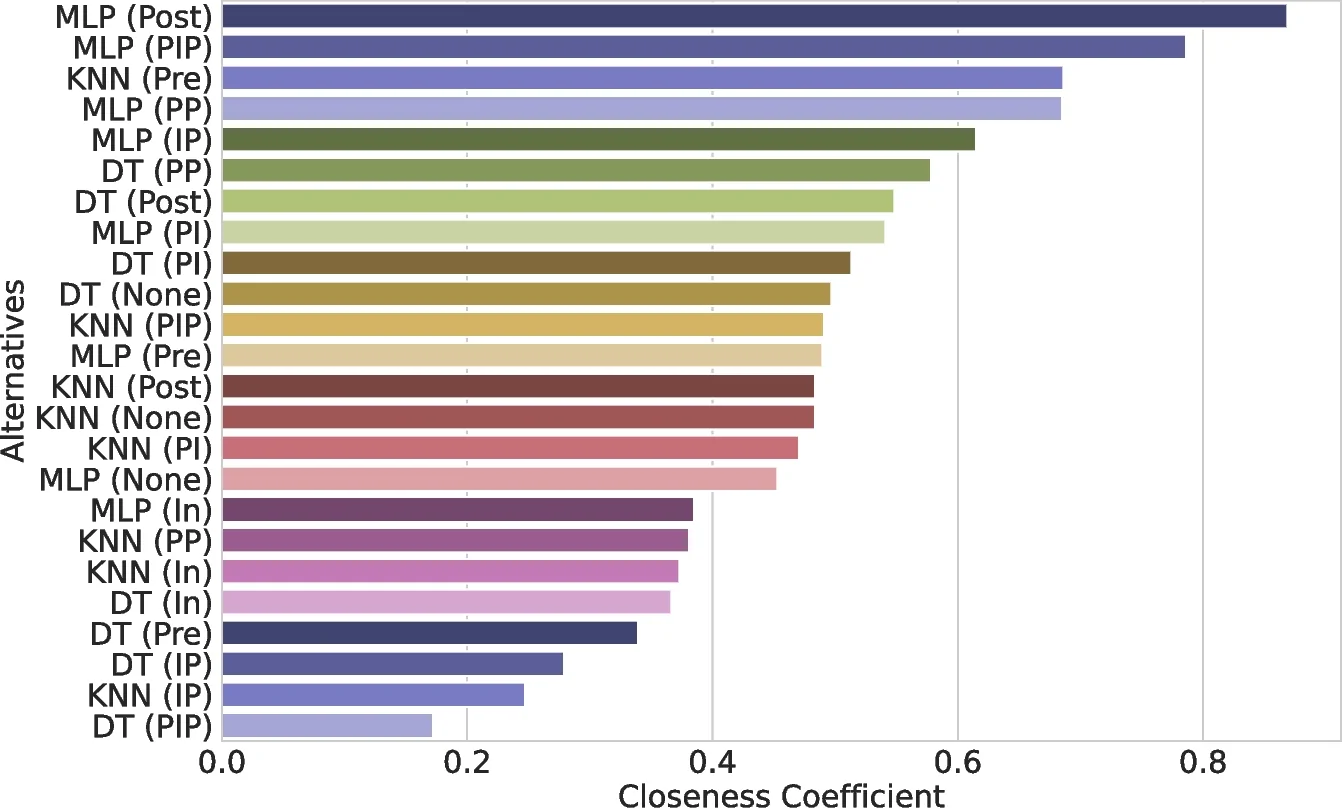
Visualization of the A-TOPSIS rankings for the DB-HIBA dataset

Figure 7 shows the visualization of the rankings obtained for each model and classifier combination, based on the proximity coefficients calculated by the A-TOPSIS algorithm applied to the DB-HIBA dataset. The results indicate that the best configuration was the *Post* approach with the MLP classifier, followed by the combination of the three approaches (*PIP*), also with the MLP classifier. The *Pre* approach with the KNN classifier was ranked third.

These results once again highlight the existence of a clear trade-off between performance metrics and fairness metrics. The approach that achieved the highest balanced accuracy (BACC) did not rank among the top ten positions in the overall ranking. In contrast, the configuration ranked first showed a *5.58%* reduction in BACC compared with its respective baseline model, which corresponded to the best overall result for this metric. Nevertheless, this same configuration achieved proximity coefficients closer to the ideal solution in the fairness metrics, particularly for Disparate Impact (DI) and Statistical Parity (SP), for which it achieved the second-best overall result. These findings justify its characterization as the best configuration for this dataset according to the criteria adopted in the conducted experiments.

Finally, for the DB-MIDAS dataset, similarly to what was observed for DB-HIBA, the best performance results were obtained by the model without the application of mitigation techniques (*None*), in this case with the KNN classifier, reaching a balanced accuracy (BACC) of *0.6987 ± 0.0102* and a recall of *0.7000 ± 0.0098*. As observed for the DB-HIBA dataset, no single approach was identified that simultaneously achieved optimal performance across all four evaluated fairness metrics.

The post-processing technique achieved the best performance for the SP metric with the MLP classifier, obtaining a percentage improvement of *93.07%* over the best baseline result. For the EOD metric, the best result was obtained by the *In + Pre* (IP) combination, also with the MLP classifier, resulting in an improvement of *33.33%* compared to the baseline. The DI metric also achieved its best result with the IP approach, this time with the DT classifier, with a gain of *17.47%* relative to the baseline. Finally, the AOD metric achieved its best value with the pre-processing technique, corresponding to an improvement of *21.61%* over the baseline.

**Fig. 8:**
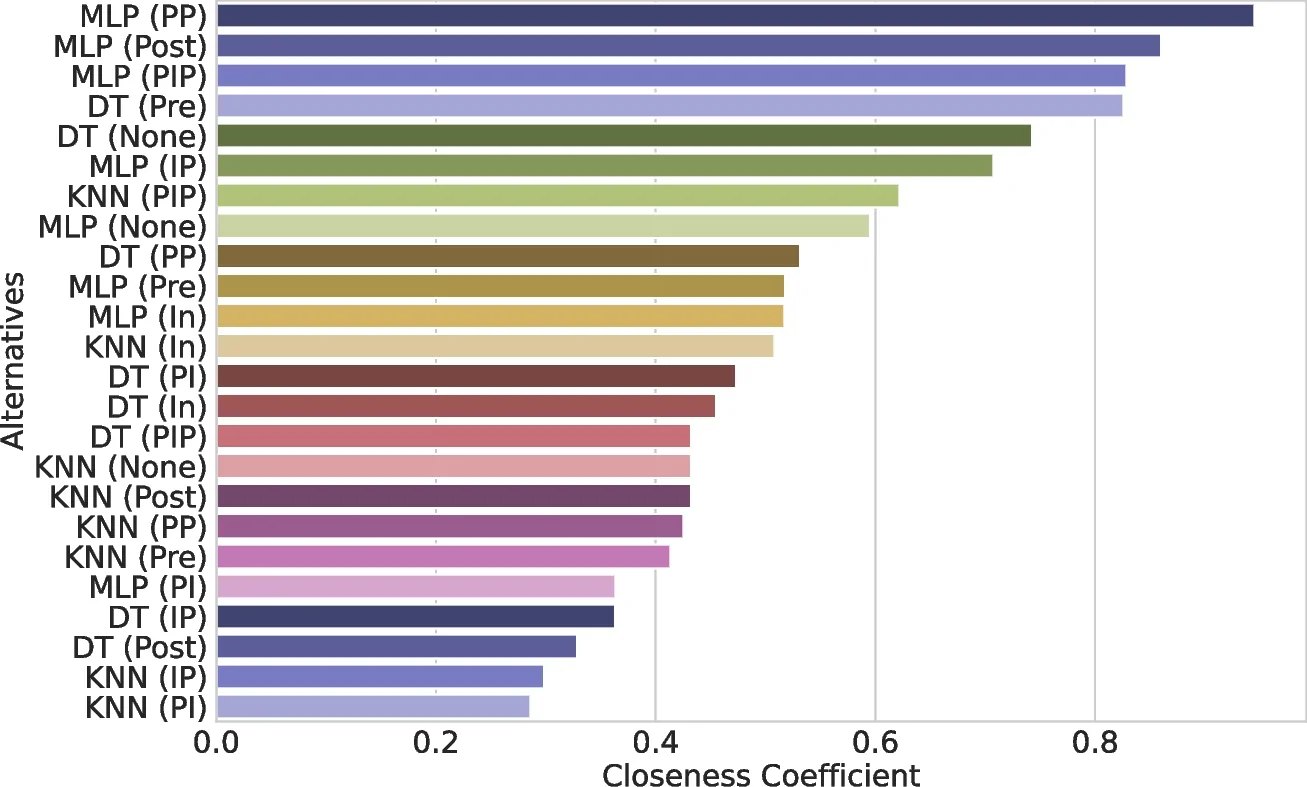
Visualization of the A-TOPSIS rankings for the DB-MIDAS dataset

Figure 8 shows the rankings of the combinations between mitigation approaches and classifiers, based on the proximity coefficients calculated via the A-TOPSIS algorithm applied to the DB-MIDAS dataset. The results indicate that the MLP classifier was present in the three highest-ranked configurations. The first position was occupied by the *Pre + Post* (PP) combination, followed by the *Post* approach. In third place was the configuration composed of the combination of the three mitigation techniques (*PIP*).

These results reinforce the trend observed in the other datasets, where bias mitigation approaches tend to show lower performance in exchange for gains in fairness metrics. Although the model without mitigation presented the best values for performance metrics, the global analysis reveals that the *Pre + Post* (*PP*) approach with the MLP classifier offers a more balanced solution. This configuration did not achieve the best absolute result in any of the fairness metrics; nevertheless, it reached the second-best overall result for the SP metric and showed a loss of only *0.014%* in BACC of the best-performing model, thereby characterizing it as the best alternative for this dataset according to the criteria defined in the experiments.

In summary, the experiments conducted confirmed some of the hypotheses formulated at the beginning of this study. The first concerns the existing trade-off between performance and fairness metrics, evidenced across all analyzed datasets. The second hypothesis regards the absence of a bias mitigation method, among those evaluated in this work, capable of simultaneously improving all metrics and presenting consistent performance across different data types such as, in our case, the use of probabilities derived from clinical or dermoscopic images in conjunction with their associated clinical data.

The variation in the performance of mitigation approaches across the DB-PAD-UFES-20, DB-HIBA, and DB-MIDAS datasets may be associated with structural differences in the sensitive data, such as the absence of patients with darker skin tones in the HIBA and MIDAS datasets. This limitation compromises the ability to properly evaluate the effectiveness of fairness techniques, particularly with respect to the Fitzpatrick variable.

From a practical standpoint, the results indicate that the adoption of post-processing techniques should be considered, as this approach was present in all configurations with the best overall performance, either in isolation or in combination, regardless of the dataset analyzed. Furthermore, combining mitigation methods, particularly at the pre-processing and post-processing stages, proved effective in promoting a balance between predictive performance and fairness in real-world clinical scenarios. However, the selection of the most appropriate mitigation method must take into account the specific characteristics of the dataset and the sensitive groups of interest.

Among the advantages of the proposed approach, the flexibility of the model proposed by Lima et al. [17] stands out. In addition to enabling the interpretability of clinical data, it can be modified to integrate different external classifiers, without being restricted to a single architecture, as well as allowing the application of mitigation techniques at multiple stages of the modeling pipeline. A relevant limitation of the approach adopted in this study lies in the requirement for additional clinical data, beyond images and sensitive variables, in order for the results to be properly computed, which are often unavailable in some datasets.

## Conclusion

This study conducts an in-depth comparison of various bias mitigation techniques applied to skin cancer datasets, with a particular focus on tabular data. By leveraging a multimodal mixed-fusion architecture, both lesion images and associated patient data can be effectively combined. Subsequently, the original PAD-UFES-20, HIBA, and MIDAS datasets were transformed, resulting in the construction of structured tabular subsets named DB-PAD-UFES-20, DB-HIBA, and DB-MIDAS. We investigated mitigation approaches across Pre-processing, In-processing, and Post-processing stages, as well as their combinations, in conjunction with three classifiers. By integrating these strategies at multiple stages of the machine learning pipeline and evaluating them through the ranking algorithm A-TOPSIS. This study establishes a methodological foundation for future research aimed at developing fairer and more reliable clinical decision-making models. According to the obtained results, it was observed that post-processing techniques were present in all top-performing configurations, either alone or in combination, suggesting their relevance regardless of the dataset. These findings provide baselines for future research on algorithmic fairness in automated skin cancer diagnosis, paving the way for the investigation of bias mitigation techniques that combine imaging and tabular data. A limitation of this study is that we examined only one bias mitigation method at each stage, while other methods available in the literature could also be explored. As future work, we propose the investigation of mitigation techniques applicable to other types of data.

## Data Availability

The data that support the findings of this study are available from the corresponding author upon reasonable request.

## References

[1] WHO (2020) WHO report on cancer: setting priorities, Investing Wisely and Providing Care for All. World Health Organization, Geneva

[2] National Institute of Cancer José Alencar Gomes da Silva (INCA) (2023) Cancer incidence in Brazil - 2023 Estimates. Available at https://www.inca.gov.br/sites/ufu.sti.inca.local/files/media/document/estimativa-2023.pdf

[3] Ferreira FRT, Couto LM, Matos MA, Oliveira Ribeiro Pinto JV, Pensabem GR (2025) Brain Tumor detection and classification in magnetic resonance imaging using AT-YOLOv10 based on Mobilenetv3 and NAM. Clust Comput 28(11):715

[4] Ferreira FRT, Couto LM (2025) Using deep learning on microscopic images for white blood cell detection and segmentation to assist in leukemia diagnosis. J Supercomput 81(2):410

[5] Rocha MB, Pratavieira S, Vieira RS, Geller JD, Stein ALM, de Oliveira FSS, Canuto TRP, de Paula Vieira L, Rossoni R, Santos MCS, Frasson PHL, Krohling RA (2025) Fluorescence images of skin lesions and automated diagnosis using convolutional neural networks. Photodiagn Photodyn Ther 52:104462 10.1016/j.pdpdt.2024.104462 39736369

[6] Pacheco AG, Krohling RA (2020) The impact of patient clinical information on automated skin cancer detection. Comput Biol Med 116:103545 10.1016/j.compbiomed.2019.103545 31760271

[7] Lima LM, Assis MCFR, Soares JP, Grão-Velloso TR, Barros LAP, Camisasca DR, Krohling RA (2023) Importance of complementary data to histopathological image analysis of oral leukoplakia and carcinoma using deep neural networks. Intell Med 03(04):258–266

[8] Lima LM, Krohling RA (2022) Exploring advances in transformers and CNN for skin lesion diagnosis on small datasets. Brazilian Conference on Intelligent Systems. Springer International Publishing, pp 282–296

[9] Rezk E, Eltorki M, El-Dakhakhni W (2023) Interpretable skin cancer classification based on incremental domain knowledge learning. J Healthcare Inf Res 7(1):59–83 10.1007/s41666-023-00127-4 PMC9995827 36910915

[10] Song B, Zhang C, Sunny S, Kc DR, Li S, Gurushanth K, Mendonca P, Mukhia N, Patrick S, Gurudath S, Raghavan S, Tsusennaro I, Leivon ST, Kolur T, Shetty V, Bushan V, Ramesh R, Pillai V, Wilder-Smith P, Suresh A, Kuriakose MA, Birur P, Liang R (2023) Interpretable and reliable oral cancer classifier with attention mechanism and expert knowledge embedding via attention map. Cancers 15(5):1421 10.3390/cancers15051421 PMC10001266 36900210

[11] Xua B, Yang G (2024) Interpretability research of deep learning: a literature survey. Inf Fusion 115:102721

[12] Assis A, Dantas J, Andrade E (2024) The performance-interpretability trade-off: a comparative study of machine learning models. J Reliable Intell Environ 11(1):1

[13] Ferreira FRT, Couto LM (2025) Explainable machine learning framework integrating PPPM for automated cytological triage and personalized breast cancer risk stratification. Netw Model Anal Health Inf Bioinform 14(1):167

[14] Guarrasi V, Aksu F, Caruso CM, Di Feola F, Rofena A, Ruffini F, Soda P (2025) A systematic review of intermediate fusion in multimodal deep learning for biomedical applications. Image Vis Comput 158:105509

[15] Boulahia SY, Amamra A, Madi MR, Daikh S (2021) Early, intermediate and late fusion strategies for robust deep learning-based multimodal action recognition. Mach Vis Appl 32(6):121

[16] Teoh JR, Dong J, Zuo X, Lai KW, Hasikin K, Wu X (2024) Advancing healthcare through multimodal data fusion: a comprehensive review of techniques and applications. PeerJ Comput Sci 10:2298 10.7717/peerj-cs.2298 PMC11623190 39650483

[17] Lima LM, Rocha MB, Krohling RA (2026) A novel interpretable approach to deep multimodal data fusion applied to cancer diagnosis. In: Freitas R, Furtado D (eds) Intelligent Systems. Springer, Cham, pp 35–49

[18] Hort M, Chen Z, Zhang JM, Harman M, Sarro F (2024) Bias mitigation for machine learning classifiers: a comprehensive survey. ACM J Responsib Comput 1(2):1–52

[19] Parraga O, More MD, Oliveira CM, Gavenski NS, Kupssinskü LS, Medronha A, Moura LV, Simões GS, Barros RC (2025) Fairness in deep learning: A survey on vision and language research. ACM Comput Surv 57(6):1–40

[20] Saeidnia HR, Hosseini E, Lund B, Tehrani MA, Zaker S, Molaei S (2025) Artificial intelligence in the battle against disinformation and misinformation: a systematic review of challenges and approaches. Knowl Inf Syst 67(4):3139–3158

[21] Raposo VL (2024) When facial recognition does not ‘recognise’: erroneous identifications and resulting liabilities. AI & SOCIETY 39(4):1857–1869

[22] Koçak B, Ponsiglione A, Stanzione A, Bluethgen C, Santinha J, Ugga L, Huisman M, Klontzas ME, Cannella R, Cuocolo R (2025) Bias in artificial intelligence for medical imaging: fundamentals, detection, avoidance, mitigation, challenges, ethics, and prospects. Diagn Interv Radiol 31(2):75–88 10.4274/dir.2024.242854 PMC11880872 38953330

[23] Aayushman, Gaddey H, Mittal V, Chawla M, Gupta GR (2024) Fair and Accurate Skin Disease Image Classification by Alignment with Clinical Labels. In: Proceedings of medical image computing and computer assisted intervention – MICCAI 2024. Springer, Marrakesh, Morroco, vol LNCS 15001, pp 394–404

[24] Park J, Lee S, Lee Y (2025) FairDITA: disentangled image-text alignment for fair skin cancer diagnosis. Journal of Imaging Informatics in Medicine 1–16 10.1007/s10278-025-01693-2 PMC13230438 41003966

[25] Corbin A, Marques O (2023) Assessing bias in skin lesion classifiers with contemporary deep learning and post-hoc explainability techniques. IEEE Access 11:78339–78352

[26] Paxton K, Aslansefat K, Thakker D, Papadopoulos Y, Maslekar T (2026) Enhancing fairness in skin lesion classification for medical diagnosis using prune learning. IEEE J Biomed Health Inform 30(8):6605–6614 10.1109/JBHI.2026.3652910 41525640

[27] Dang VN, Campello VM, Hernández-González J, Lekadir K (2025) Empirical comparison of post-processing debiasing methods for machine learning classifiers in healthcare. J Healthcare Inf Res 9(3):465–493 10.1007/s41666-025-00196-7 PMC12290158 40726749

[28] Feldman T, Peake A (2021) End-to-end bias mitigation: removing gender bias in deep learning. arXiv:2104.02532 arXiv preprint

[29] Zong Y, Yang Y, Hospedales T (2023) MEDFAIR: benchmarking fairness for medical imaging. In: International conference on learning representations

[30] Bayasi N, Fayyad J, Hamarneh G, Garbi R, Najjaran H (2025) Debiasify: self-distillation for unsupervised bias mitigation. In: 2025 IEEE/CVF winter conference on applications of computer vision (WACV), pp 3227–3236

[31] Goswami M, Chatterjee R, Sekh AA (2026) FairMed-XGB: a bayesian-optimised multi-metric framework with explainability for demographic equity in critical healthcare data. arXiv:2603.14947 arXiv preprint

[32] Krohling RA, Pacheco AGC (2015) A-TOPSIS - an approach based on TOPSIS for ranking evolutionary algorithms. Proc Comput Sci 55:308–317. 3rd International Conference on Information Technology and Quantitative Management, ITQM 2015

[33] Xu Z, Li J, Yao Q, Li H, Zhao M, Zhou SK (2024) Addressing fairness issues in deep learning-based medical image analysis: a systematic review. NPJ Digit Med 7(1):286 10.1038/s41746-024-01276-5 PMC11487181 39420149

[34] Gulamali F, Sawant AS, Liharska L, Horowitz C, Chan L, Hofer I, Singh K, Richardson L, Mensah E, Charney A, Reich D, Hu J, Nadkarni G (2025) Detecting, characterizing, and mitigating implicit and explicit racial biases in health care datasets with subgroup learnability: Algorithm development and validation study. J Med Int Res 27:71757 10.2196/71757 PMC12410029 40905712

[35] Lee MS, Guo LN, Nambudiri VE (2022) Towards gender equity in artificial intelligence and machine learning applications in dermatology. J Am Med Inform Assoc 29(2):400–403 10.1093/jamia/ocab113 PMC8757299 34151976

[36] Dehghani F, Paiva P, Malik N, Lin J, Bayat S, Bento M (2025) Accuracy-fairness trade-off in ml for healthcare: A quantitative evaluation of bias mitigation strategies. Inf Softw Technol 188:107896

[37] Luo H, Zhou Z, Shu SZ, Mortanges APd, Berke R, Reyes M (2026) On the interplay of human-ai alignment, fairness, and performance trade-offs in medical imaging. In: Gee JC, Alexander DC, Hong J, Iglesias JE, Sudre CH, Venkataraman A, Golland P, Kim JH, Park J (eds) Medical image computing and computer assisted intervention – MICCAI 2025. Springer, Cham, pp 420–430

[38] Kamiran F, Calders T (2009) Classifying without discriminating. In: 2nd International conference on computer, control and communication, pp 1–6

[39] Calders T, Kamiran F, Pechenizkiy M (2009) Building classifiers with independency constraints. In: IEEE international conference on data mining workshops, pp 13–18

[40] Kamiran F, Calders T (2012) Data preprocessing techniques for classification without discrimination. Knowl Inf Syst 33(1):1–33

[41] d’Aloisio G, D’Angelo A, Di Marco A, Stilo G (2023) Debiaser for Multiple Variables to enhance fairness in classification tasks. Inf Process Manag 60(2):103226

[42] Dwork C, Hardt M, Pitassi T, Reingold O, Zemel R (2012) Fairness through awareness. In: Proceedings of the 3rd innovations in theoretical computer science conference. ITCS ’12. Association for Computing Machinery, New York, NY, USA, pp 214–226

[43] Beutel A, Chen J, Zhao Z, Chi EH (2017) Data decisions and theoretical implications when adversarially learning fair representations. arXiv:1707.00075 arXiv preprint

[44] Kamishima T, Akaho S, Sakuma J (2011) Fairness-aware learning through regularization approach. In: IEEE 11th international conference on data mining workshops, pp 643–650

[45] Zheng G, Jacobs MA, Braverman V, Parekh VS (2025) Towards fair medical AI: adversarial debiasing of 3D CT foundation embeddings. arXiv:2502.04386 arXiv preprint

[46] Penz D, Escobedo Ticona GJ, Schedl M (2025) Mitigating latent user biases in pre-trained vae recommendation models via on-demand input space transformation. Proceedings of the Nineteenth ACM Conference on Recommender Systems. RecSys ’25. Association for Computing Machinery, New York, NY, USA, pp 632–636

[47] Calders T, Verwer S (2010) Three naive bayes approaches for discrimination-free classification. Data Min Knowl Disc 21(2):277–292

[48] Pedreschi D, Ruggieri S, Turini F (2009) Measuring discrimination in socially-sensitive decision records. In: Proceedings of the 2009 SIAM international conference on data mining (SDM), pp 581–592

[49] Alabdulmohsin IM, Lucic M (2021) A Near-Optimal Algorithm for Debiasing Trained Machine Learning Models. In: Ranzato M, Beygelzimer A, Dauphin Y, Liang PS, Vaughan JW (eds) Advances in Neural Information Processing Systems, vol 34. Curran Associates Inc, Canada, pp 8072–8084

[50] Pacheco AGC, Lima GR, Salomão AS, Krohling B, Biral IP, de Angelo GG, Alves Jr FCR, Esgario JGM, Simora AC, Castro PBC, Rodrigues FB, Frasson PHL, Krohling RA, Knidel H, Santos MCS, do Espírito Santo RB, Macedo TLSG, Canuto TRP, de Barros LFS (2020) PAD-UFES-20: a skin lesion dataset composed of patient data and clinical images collected from smartphones. Data in Brief 32:106221 10.1016/j.dib.2020.106221 PMC7479321 32939378

[51] Ricci Lara MA, Rodríguez Kowalczuk MV, Lisa Eliceche M, Ferraresso MG, Luna DR, Benitez SE, Mazzuoccolo LD (2023) A dataset of skin lesion images collected in Argentina for the evaluation of AI tools in this population. Sci Data 10(1):712 10.1038/s41597-023-02630-0 PMC10584927 37853053

[52] Chiou AS, Omiye JA, Gui H, Swetter SM, Ko JM, Gastman B, Arbesman J, Cai ZR, Gevaert O, Sadée C, Rotemberg VM, Han SS, Tschandl P, Dickman M, Bailey E, Bae G, Bailin P, Boldrick J, Yekrang K, Caroline P, Hanna J, Kurtansky NR, Weber J, See NA, Phung M, Gallegos M, Daneshjou R, Novoa RA (2025) Multimodal image dataset for AI-based skin cancer (MIDAS) benchmarking. NEJM AI 2(6):2400732 10.1056/AIdbp2400732 42932542

[53] Pedregosa F, Varoquaux G, Gramfort A, Michel V, Thirion B, Grisel O, Blondel M, Prettenhofer P, Weiss R, Dubourg V, Vanderplas J, Passos A, Cournapeau D, Brucher M, Perrot M, Duchesnay E (2011) Scikit-learn: machine learning in Python. J Mach Learn Res 12:2825–2830

[54] Akiba T, Sano S, Yanase T, Ohta T, Koyama M (2019) Optuna: a next-generation hyperparameter optimization framework. In: Proceedings of the 25th ACM SIGKDD international conference on knowledge discovery & data mining. Association for Computing Machinery, New York, NY, USA, pp 2623–2631

[55] Bergstra J, Bardenet R, Bengio Y, Kégl B (2011) Algorithms for hyper-parameter optimization. In: Proceedings of the 24th international conference on neural information processing systems. NIPS’11. Curran Associates Inc, Red Hook, NY, USA, pp 2546–2554

[56] Saxena NA, Huang K, DeFilippis E, Radanovic G, Parkes DC, Liu Y (2020) How do fairness definitions fare? Testing public attitudes towards three algorithmic definitions of fairness in loan allocations. Artif Intell 283:103238

